# Effects of forest management and roe deer impact on a mountain forest development in the Italian Apennines: A modelling approach using LANDIS-II

**DOI:** 10.1371/journal.pone.0224788

**Published:** 2019-11-06

**Authors:** Andrea Marcon, David J. Mladenoff, Stefano Grignolio, Marco Apollonio

**Affiliations:** 1 Department of Veterinary Medicine, University of Sassari, Sassari, Italy; 2 Department of Forest & Wildlife Ecology, University of Wisconsin-Madison, Russell Labs, Madison, Wisconsin, United States of America; University of Ferrara, ITALY

## Abstract

Forest development is a complex phenomenon which, for the number of actors involved and the response time expressed by forests, is difficult to understand and explore. Forests in Italy, as in several areas of Europe, are experiencing intensive management and recently, an increasing impact by ungulates. The effects on forest development of these two disturbances combined are difficult to predict, and consequently to be properly managed. We used a forest landscape change model, LANDIS-II, to simulate forest development as driven by forestry practices and roe deer impact for 200 years in a mountain forest of the Italian Apennines. We found that each disturbance alters forest tree species richness, forest type abundance and distribution, and forest structure. When considered combined, the two disturbances show additive behavior, enhancing or moderating each other’s effects. Forest management has a negative effect on tree species richness. We expected roe deer to have a negative effect on harvest yields, but this result was significant only for two of seven harvesting treatments. On the other hand, roe deer presence had a positive effect on tree species richness. All the simulation scenarios returned some extent of forest loss. The amount of the forest loss is lowest in the scenario without disturbances, and greatest when both disturbances are considered. However, the two disturbances combined, with the magnitude modelled in our simulations, have relatively low effects on the forest dynamics we analyzed in our study area. LANDIS-II was an effective approach for simulating combined management and ungulate driven trends of forest development, and to help understand the dynamics that lay behind it.

## Introduction

Human activities are the primary cause of forest change in wide areas of the world [1]. Since the beginning of the agricultural phase in human history, the need for wood and rangelands to raise livestock drove humans to shape the extent and composition of the forest to match their needs. In the last centuries, human population increase has caused an even stronger effect on forest extent and composition, causing—in some areas of world–the development of completely artificial forests [2–4]. Wild ungulate populations often faced a rapidly changing environment, different from the one they evolved in. As long as hunting and habitat reduction kept wild ungulate populations at low densities in Western countries, their effect on forest development was usually low. In the last half-century, however, wild ungulate populations have strongly increased their number, especially in Europe, North America and Japan [5–9], becoming environmental engineers strongly able to shape forest structure and development [10–14]. Developing a better understanding of how human activities and ungulate impacts interact with each other and influence the development of the forests is of major concern for forest managers, game managers, conservationists and other stakeholders. The pressure of these two disturbances can affect biodiversity, natural resource sustainability and important economic use of the forest [15–18]. Species composition, stand structure, and landscape heterogeneity, often artificially created by forest management, can have a strong influence on shaping ungulate impact, which in turn modifies the characteristics of the vegetation of the area they live in [17,19–24]. Many studies have been devoted to the impact of ungulate populations on forests, with special reference to high densities of ungulates [25–28]. Those studies, however, focused mostly on the immediate impact of ungulates, while only a few of them on the long-term effects, such as changes in tree species abundance and distribution, tree species richness, and forest structure [29–31].

Forest development is a complex ecological process, in which multiple factors interact at different scales. To understand and simulate those complex dynamics, several types of forest ecosystem models have been developed and used in ecology [32–35], which focus on different aspects of the same phenomenon. The main aim of our study is to simulate the effects of two disturbances (forest management and roe deer *Capreolus capreolus* impact, i.e. the most widespread ungulate in Europe) on forest structure, composition, tree species richness and extent of the forested area in a heavily managed forest, including three protected areas where harvesting is subject to some limitations. The outputs of our model simulation can be used to understand interactions between disturbances, and to identify the ecological trends that would emerge from those interactions. We simulated four scenarios of forest development, initially without any disturbance, and afterwards including the two disturbances “Harvesting” and “Roe deer”, considered both individually and combined. The simulations have been performed using LANDIS-II (LANDscape DIsturbance and Succession) model framework, which simulates the development of forested landscapes taking into account ecological processes, such as succession, seed dispersal, harvesting, and a set of biotic and abiotic disturbances [36]. LANDIS-II is a process-based and spatially explicit model framework based on the original LANDIS model [37,38], which operates on raster maps, where every cell contains information about tree species, ecological variables and disturbances. It is ideally suited to our research questions, as it models multiple ecological and anthropogenic processes such that the interactions of these processes are an emergent property of the simulations [39]. This software has been used to explore forest landscape dynamics in many parts of the world [36,40–43], but, to our knowledge, only once considering ungulates [30]. To summarize, the questions examined in the present study are: i) are silvicultural treatments and ungulate disturbance interacting? ii) is one of the two disturbances leading the shaping of forest development? iii) can analyses of disturbances’ effects, performed through process modelling, help in the development of management policies?

## Methods

### Study area

**Fig 1:**
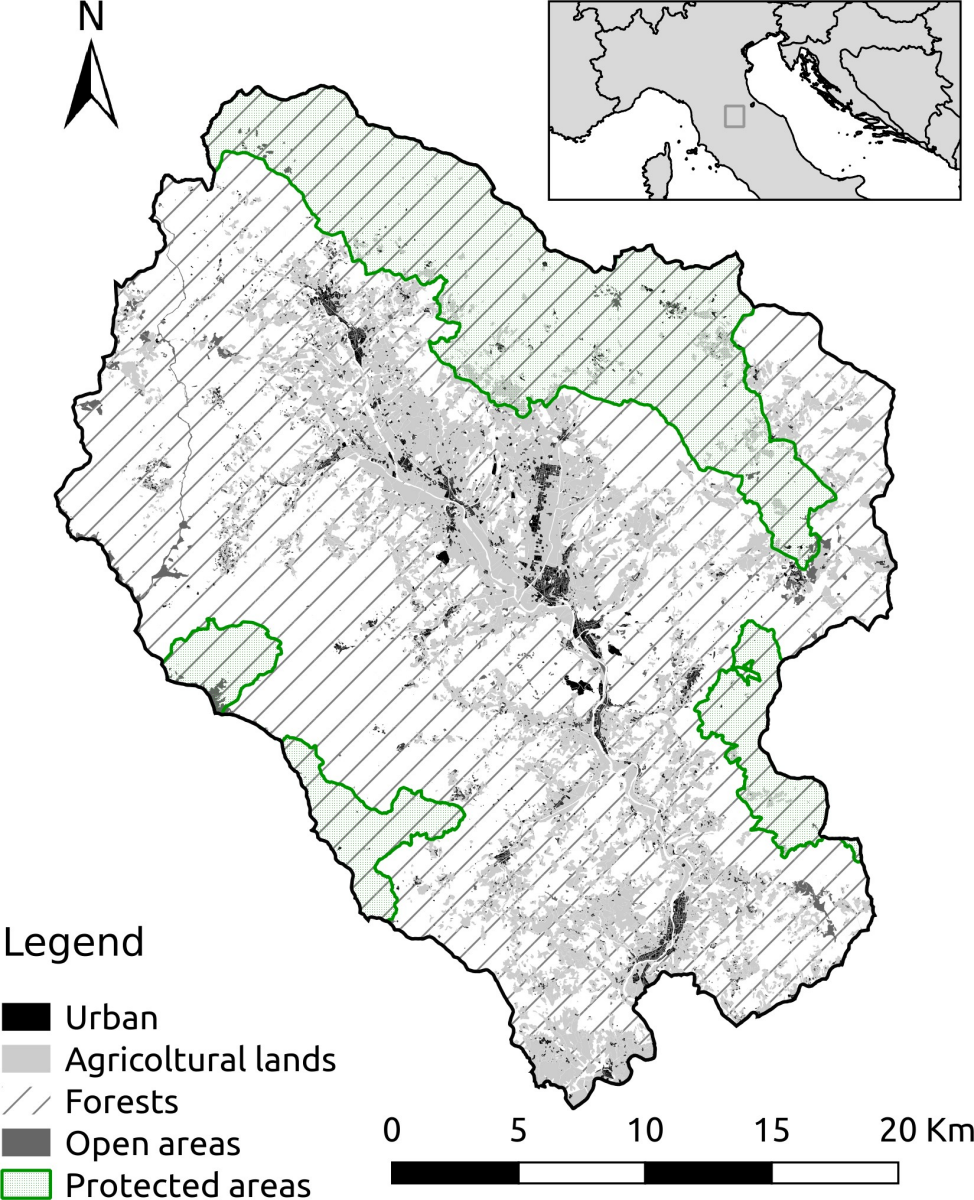
Study area, Casentino valley, in Arezzo province, Tuscany, Italy.

The study was carried out in the Casentino valley (43°43'46.3"N, 11°46'21.8"E), in Arezzo Province, Tuscany, Italy (Fig 1), a mountainous region of the northern Apennines, ranging in elevation from 200m to 1655m a.s.l. The climate is temperate (Cfc in Köppen classification), characterized by hot and dry summers and cold and rainy winters, with a high humidity rate. Due to the wide altitude range covered, mean temperatures range widely between the mountain tops and the valleys. The mean January and July temperatures are reported for both highest and lowest areas, respectively: January 1.3°C and 4.2°C; July 19.3°C and 22.5°C. The mean annual precipitation range (2000–2012), was from 900 mm to 1500 mm. The area has an extent of 82’614 ha, 67.6% of it is covered by forest, while urban areas cover only 1% of the surface. Agricultural lands represent a small percentage of the area and are mostly found close to human settlements at the lowest altitudes toward the study area center. Part of a national park and two protected areas are included in the study area: Foreste Casentinesi National Park (13’845 ha in the study area), Pratomagno OAF (5’379 ha) and Alpe di Catenaia OAF (2’760 ha). Until the mid 1960s, this area was heavily used by humans, mostly with small scale agriculture and livestock grazing. Forest patches could be found only at highest elevations, in areas less suited for agriculture. After that period most of the human population moved towards lowlands and cities, and land use shifted towards forestry. Nowadays, almost all the forests in the study area are managed, mostly as coppice. Other management techniques applied are selection cutting and thinning, mostly in the protected areas. At the lowest elevations, mixed broadleaf forest patches dominate the landscape, and at mid-elevations some conifers patches, due to artificial afforestation in the past, are present. At the highest elevation, the forest is largely composed of beech (*Fagus sylvatica*). The most abundant broadleaf species are oaks (*Quercus* spp.), beech, chestnut (*Castanea sativa*), and hornbeam (*Ostrya carpinifolia*), while conifers are white fir (*Abies alba*), black pine (*Pinus nigra*), exotic Douglas fir (*Pseudotsuga menziesii*), and exotic maritime pine (*Pinus pinaster*). Four ungulate species are present in the study area, two are widely abundant, roe deer and wild boar (*Sus scrofa*), while the others, red deer and fallow deer (*Cervus elaphus* and *Dama dama*) are locally abundant but more dispersed. Only roe deer has been considered in the modelling being the most abundant ubiquitous deer species (density consistently higher than 12 individuals per square kilometre, [44]). Two predators are present in the area, wolf (*Canis lupus*) and red fox (*Vulpes vulpes*).

### Model description

LANDIS-II models forest landscape change at a range of broad spatial and temporal scales. The model is spatially explicit and raster-based (i.e. the landscape is represented by a grid), and it is used to simulate the ecological dynamics involved in landscape development, by modelling processes such as forest succession and disturbances. There are several extensions that can be used to model the latter, from harvesting to windthrow to biological disturbances. The software allows the user to choose between different level of modelling complexity, based on the starting data available and the research questions. A detailed description of the LANDIS-II model framework can be found in the literature ([36,39,45] http://www.landis-ii.org). We choose to model forest development for 200 years, with a 3-year time step. We thought this time span long enough to identify emerging ecological trends, whereas modelling the forest for a longer time span (i.e. 800 years), while ignoring the climatic and socio-economical changes that would occur in that time span, would probably return a simulation so disconnected from the real course of events that the meaning of the results would be limited. For our study, we chose to use the Age Only Succession v.3.0 extension, which models the tree species as presence or absence of species and their age cohorts (i.e. group of homogeneous ages, e.g. 0–3 years, 4–6 years, etc.), inside each cell of the grid that represents the study area. To model forest management and roe deer impact we used the Base Harvest v.2.2 extension [46]. We defined 4 different scenarios, to compare the effects of disturbances on forest development and composition. The scenarios are: No Disturbance (ND), Harvesting (H), Roe Deer (RD), and Harvesting & Roe Deer (HRD) combined. All spatial data handling was done with ArcGIS v.10 (ESRI) and all data analysis with R v.3.0 [47].

### Datasets

Species assemblages and dominance have been extracted from a regional dataset (Tuscan Forest Survey, *Inventario Forestale Toscano*, IFT, Regione Toscana, 1998). From this grid-structured dataset, with 400m cells, we extracted data on the canopy cover of the cells (as percentage), and the three most abundant tree species ranked in order of abundance. Tree species physiological data (see paragraph Ecoregion map) have been collected from literature [48–62] and extracted from TRY database (the TRY initiative [63]). Soil composition data and soil water content have been extracted from the pedological dataset of the watershed of the Arno river [64]. Climatic data have been extrapolated from data sets granted by Corpo Forestale dello Stato (Pratovecchio and Pieve Santo Stefano stations). Ungulate distribution data have been collected by the Arezzo URCA (Apennine Hunting Association) and validated by the Wildlife and Hunting Department of Arezzo Province. Roe deer impact data have been extracted from literature, from studies conducted in the same area [65–67]. These studies analyzed roe deer impact on oak and chestnut coppice areas on a long-term monitoring, and along a gradient of roe deer densities. Those findings were corroborated and expanded by the forest managers of the area, leading to the identification of the following preferred species: *Castanea sativa*, *Fagus sylvatica*, *Quercus cerris*, *Quercus pubescens*, *Abies alba*, *Acer pseudoplatanus*, *and Fraxinus ornus*.

### Input data

#### Initial communities map

**Fig 2:**
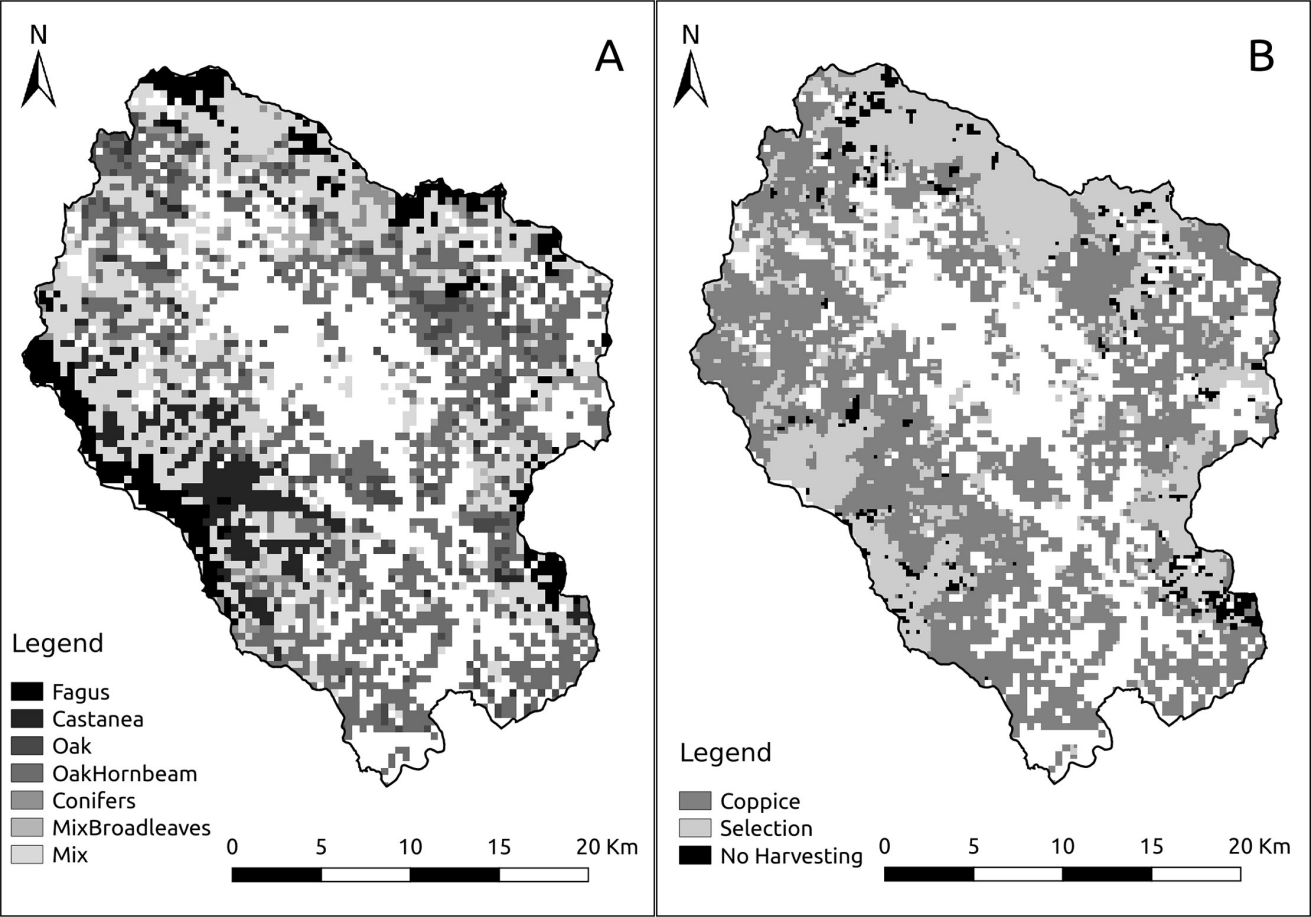
Spatial distribution of (A) major forest types in the study area at the beginning of the simulation, and (B) of forest management areas. White grid cells inside the study area are not forested cells (i.e. canopy cover <5%).

Our original tree species dataset was too coarse for the extent of our study area (400 x 400m cells), so we downscaled the data halving the cell size. The data contained in each of the four new cells were copied directly from the original cell. The downscale did not improve the initial dataset precision but was performed to allow a higher resolution expression of the simulated dynamics. As LANDIS-II is spatially explicit, what surrounds a certain cell influences its future state. Large cells might hinder small-scale processes, e.g. the seeding dispersal, and prevent the expression of some dynamics. For this reason, we decided to halve the cell side dimension, to obtain a a higher number of smaller cells that would better represent the heterogeneity of the modelled dynamics at the end of the simulations. We use the canopy cover data in the IFT to exclude from the modelling those cells that were not forested (overall canopy cover <5%, white cells in the maps, Fig 2). To select the tree species for the modelling, we used the presence data for each species, weighted for the rank they have in each cell, to calculate an index of abundance. Calculating the cumulative percentage of tree species abundance, we found that 9 species account for more than 97% of the overall forest cover, those species are: *A*. *alba*, *C*. *sativa*, *F*. *sylvatica*, *O*. *carpinifolia*, *P*. *nigra*, *P*.*s pinaster*, *P*. *menziesii*, *Q*. *cerris*, and *Q*. *pubescens*. We add to those 9 species another 3 minor species, *Acer pseudoplatanus*, *Fraxinus ornus* and *Robinia pseudoacacia*, the formers because are highly preferred by ungulates (e.g. [68]) and the latter because it’s an alien species considered a pest. *R*. *psuedoacacia* is considered to be browsed by deer species (e.g. [69]), but this does not seem to apply to our study area. The life traits of the species can be found in Table A in S1 File. The Ward algorithm has been used to model seed dispersal, as a probability distribution that follows a negative exponential curve for both the effective and maximum seeding distances [70]. We used the information on the ranking of the three most abundant species inside each cell to extrapolate the most representative species assemblages (forest types, see Fig 2A), with an abundance driven method. Tree species age data have been extrapolated from the Forestry Management Plans produced by Casentino Municipality Union, the institution that manages the regional owned forests. For each species of our cells, we assigned the age recorded for the closest parcel (i.e. small areas that are considered a unit in the Forestry Management Plan).

#### Ecoregions map

The model also requires an ecoregion map, a raster map that identifies ecologically homogeneous regions in the study area. Ecologically homogeneous here refers to those ecological conditions that influence tree species establishment. To identify these ecoregions we used a clustering technique on a dataset containing 6 variables: altitude, available water content, soil pH in the firsts 50 cm, PAR (Photosynthetic Active Radiation), organic matter content in the firsts 50 cm, and average annual precipitations. We apply a PAM clustering method (Partitioning Around Medoids [71], package “cluster” [72]) with several different *k* parameters. Then we assessed the most reliable value for *k* through bootstrapping and then calculated the mean Jaccard index for each cluster of each *k* value [73]. The highest *k* values with all clusters having a mean Jaccard index value higher than 0.75 has been chosen. That analysis led to 5 clusters, which in turn led to 5 ecoregions.

LANDIS-II requires a probability of establishment value for each species in each ecoregion. To calculate those values, we used the software PnET-II for LANDIS-II [74,75]. This software uses ecological characteristics of a region and physiological parameters of the tree species to calculate the probability of the species to establish in that region. The physiological parameters needed by PnET-II for LANDIS-II to calculate tree species establishment probabilities are the coefficient for photosynthesis reduction due to vapor pressure deficit (VPD), the coefficient for water-use efficiency (WUE) as a function of VPD, and the minimum and maximum growing degree days for each species. For a complete list of all the parameters needed by PnET-II for LANDIS-II see Table I in S1 File. We use the medoids of our clusters as ecological data representative of each ecoregion, and calculate the monthly mean data for temperature, precipitation and PAR, starting from a 20 years-long daily raw dataset for temperature and precipitation, and GIS elaborations on a DEM file for PAR data. As PnET-II for LANDIS-II is a model developed for the eastern U.S., we asked the forest manager of our study area to check the establishment probabilities for our species. They slightly modified some of the values, for a better representation of the local situation. The establishment probability values can be found in Table B in S1 File.

#### Harvesting prescriptions

The Base Harvest extension needs a Management Areas map, a raster map that identifies areas on which the same set of harvesting prescriptions are applied (Fig 2B). Management areas have been extrapolated from the parcel map of the study area. Each parcel contains information about tree species presence, the prevalent age and the type of management applied. The most widely applied management method is coppice, especially by private owners, while in regional or state-owned forests, thinning and selection cutting prescriptions are applied (Fig 2B). Based on that, we divided our study area into three management areas: Coppice management area, Selection management area (with selection cutting and thinning prescriptions), and No Harvest management area. We defined several prescriptions to simulate the forest management practice. Those prescriptions target certain age cohorts of certain species, on the basis of the type of management practice they simulate. The harvesting prescriptions have been defined and calibrated to simulate a static application of current forest management prescriptions, and to fulfill the present forest management objectives. A list of the prescriptions applied can be found in Table C in S1 File.

The same extension has been used to model roe deer impact on forest development. The prescription that models roe deer impact targets the youngest age cohorts of its preferred tree species. Base Harvest v.2.2 prescriptions works by removing completely the targeted species’ age cohorts. The target age cohorts of that prescription are the youngest, from 0 to 6 years for the most affected species, and from 0 to 3 years for the least preferred species (Table C in S1 File). With roe deer present in the whole study area, this prescription is applied to 100% of the area. Base Harvest v.2.2 extension does not allow multiple prescriptions to be applied in a single cell at the same timestep. This makes sense for management prescriptions, but it is a theoretical error if we consider the roe deer prescription, i.e. cutting old pines does not prevent roe deer from feeding on young oaks in the same area. To get around this problem we added the roe deer effect to all the other prescriptions, so when a certain prescription is applied, it automatically applies the roe deer impact on the same cells.

#### Model outputs

Output Cohort Statistics v.2.1 extension has been used to produce output of maximum age across all species in each cell, species presence/absence for each cell, and total number of tree species in each cell. Through Output Age Reclass v.2.0 extension, we reclassified the raw data on species presence in each cell into forest types. This extension considers both species presence and dominance to classify a cell into user-determined groups, representing ecologically meaningful species assemblages for the area (Table 1). Base Harvest v.2.2 extension produce a table which shows, for each time step, how many cells were targeted by each prescription applied in that scenario, grouped by management area. LANDIS-II outputs consist of raster maps produced at a user-defined time step, each map containing one of the selected output information. We used a 20 years timestep for output map production. The maximum age across all species in each cell has been used to examine forest structure. Ages have been binned in 40 years wide bins (<40 years, 40–80 years,…, >200 years), and then the proportion of cells falling into each bin has been calculated. To better understand the regeneration dynamics, we made a second step analysis only on those cells which maximum age was lower than 100 years. Species presence/absence maps have been used to calculate the abundance for each species, expressed as the percentage of all active cells which contains that particular species. Species richness has been analyzed by calculating the number of cells that contains a certain number of species (range 1–5) at the end of the simulation (i.e. at year 200). The percentage cover of each forest type across the landscape has been calculated as the percentage of active cells that contains that forest type, for each output time step. The Base Harvest tables have been used to quantify the harvested extent for each prescription, allowing us to evaluate the differences in harvesting extent when roe deer was considered. Being that the roe deer impact is simulated as a prescription too, the same table allowed us to evaluate the extent of the area affected by roe deer, and the differences in impact between management areas. We compared the extent of the forested area at the beginning and at the end of the simulations, to check for forest loss.

**Table 1:** Species assemblages used to create forest types.

Forest Type	Species assemblage
Fagus	*Fagus sylvatica*
Castanea	*Castanea sativa*
Oaks	*Quercus cerris*, *Quercus pubescens*
OakHornbeam	*Quercus cerris*, *Quercus pubescens*, *Ostrya carpinifolia*
Conifers	*Abies alba*, *Pinus nigra*, *Pinus pinaster*, *Pseudotsuga menziesii*
MixBroadleaves	*Fagus sylvatica*, *Castanea sativa*, *Quercus cerris*, *Quercus pubescens*, *Ostrya carpinifolia*
Mix	*Fagus sylvatica*, *Castanea sativa*, *Quercus cerris*, *Quercus pubescens*, *Ostrya carpinifolia*, *Abies alba*, *Pinus nigra*, *Pinus pinaster*, *Pseudotsuga menziesii*

### Data analysis

Chi-square tests have been used to test the differences of forest structure and species richness among all the scenarios. When considering scenarios with harvesting management areas, we used the chi-squared test to check for the significance of differences between forest structures of each management area. Friedman’s test and its *post-hoc* tests have been used to test the significance of the differences for most of the other output analyzed, as our data were neither normal nor homoscedastic. We used it to test i) differences in abundance of forest types between scenarios, ii) changes in harvesting extent with and without considering roe deer impact, iii) different roe deer impact on different management areas, and iv) differences in species abundance with and without considering roe deer presence.

Note that statistical significance here is interpreted as suggested ecological effects, not significance in the traditional sense, as is in most modelling analyses.

## Results

### Forest types

**Fig 3:**
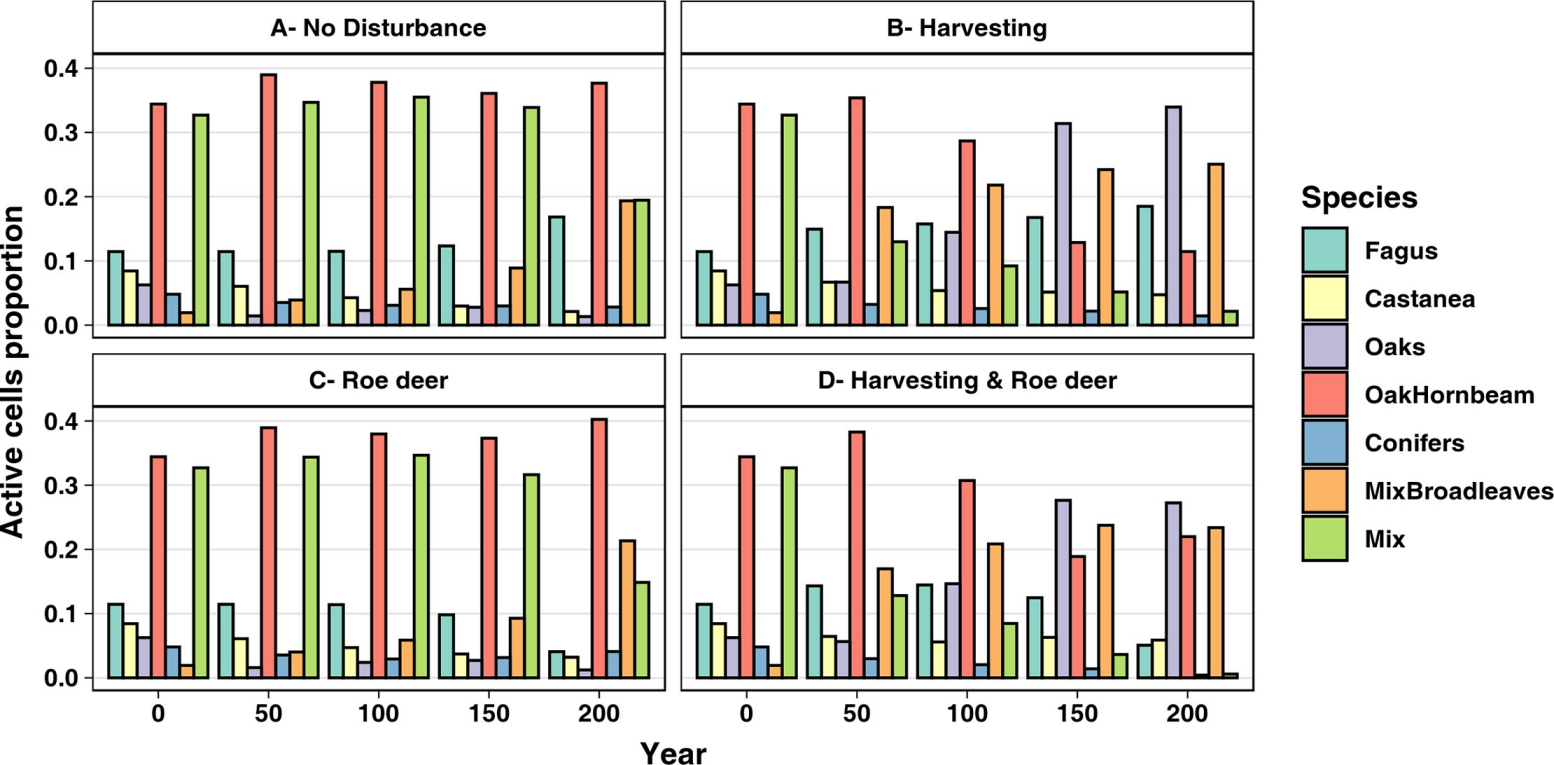
Proportions of major forest types between different scenarios, at 50-years steps.

Our simulations pointed out differences of forest types abundance among scenarios (Fig 3). The ND scenario is dominated by the Oak-Hornbeam forest type, followed by Mix, Mix Broadleaves, and Fagus forest types (37.8%, 19.5%, 19.4%, and 16.9% of the forested study area, respectively; Table D in S1 File). The remaining forest types cover around 6% of the forest area altogether. Oak-Hornbeam remained more or less stable throughout the simulation, while Fagus and Mix Broadleaves were the only types showing a constant increase (Fig 3A). The Friedman’s test and the post-hoc tests returned the difference of forest types abundance between H and ND scenarios as significant, for all the forest types (Table F in S1 File). The most abundant forest types at the end of the H scenario simulation were Oaks, Mix Broadleaves and Fagus. They were the only forest types showing a constant increasing trend throughout the simulation, ending up representing the 34.7%, 25.6%, and 18.9% of the forested study area respectively (Fig 3B). Similarly, the Friedman’s test showed significant differences in forest types abundance between RD and ND scenarios for all the forest types, except for Oaks and Conifers ones. In RD simulation, Oak-Hornbeam, Mix Broadleaves, and Mix were the most abundant forest types, covering the 45.1%, 23.9%, and 16.6% of the forested study area respectively. Different from the other scenarios, the Conifers forest type seemed to remain quite stable during the simulation, while the other forest types have decreasing trends, especially Fagus and Castanea (Fig 3C). The forest type abundance distribution estimated by the HRD scenario is significantly different from ND scenario, even though Fagus is at the significance limit, and RD scenario. When compared to H, Castanea and Oaks distributions are not significantly different, while all the other distributions are (Table F in S1 File). In HRD scenario, the most abundant forest types are Oaks, Mix Broadleaves, and Oak_Hornbeams (32.1%, 27.6%, and 25.9% of the forested study area, respectively; Table D in S1 File). Except for Oaks and Mix Broadleaves, which have an increasing trend, and Castanea, which remains quite stable, all the other forest types have decreasing trends (Fig 3D).

### Forest structure

#### Forest <100 years old

**Fig 4:**
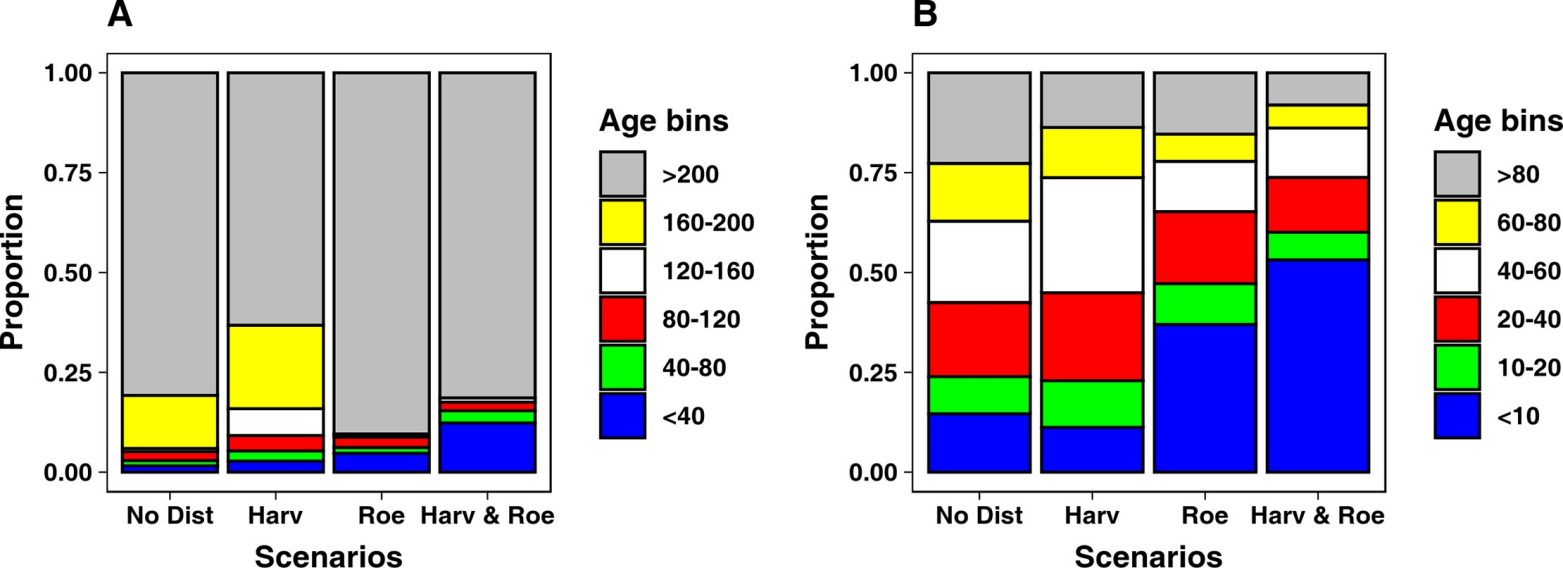
Proportion of age bin distribution between scenarios, considering all age classes (A), and only ages up to 100 years (B).

The age structure of the forest younger than 100 years is quite homogeneously distributed between age classes for both ND and H scenarios. When roe deer is considered, there is a clear shift towards younger age classes. The youngest age class (<10 years) comprises up to 53.2% of the young forest in the HRD scenario, and up to 37% in RD scenario (Fig 4B, Table E in S1 File).

#### Overall forest structure

At the end of the simulation of ND scenario the forest structure is markedly unbalanced towards older age classes (Fig 4A). The cells with maximum age over 160 years represent the 94% of the whole study area, while the younger classes cover the remaining 6%. The difference in forest structure against this scenario resulted to be statistically significant for all the other scenarios (H scenario: χ^2^_(5,N = 12)_ = 8089.8, p< 0.001, RD scenario: χ^2^_(5,N = 12)_ = 2191.0, p< 0.001, HRD scenario: χ^2^_(5,N = 12)_ = 11058, p< 0.001). The forest structure produced by the H scenario (Fig 4A) simulation is shifted to younger ages, if compared to the NB scenario. All age classes, with the exception of >200 yrs class, show an increase over the ND scenario, especially the 120–160 yrs class. Further, the cells with maximum age over 160 years comprise 84.1% of the study area. Conversely, the forest structure at the end of the RD simulation is markedly shifted towards older ages, as cells with maximum age over 200 years comprise 90.4% of the forested study area (Fig 4A). The simulations for the HRD scenario show an unbalanced structure and a prevalence of older age trees, as cells with maximum age above 200 years covers the 81.4% of the study area. On the other hand, this scenario has the highest percentage of cells which maximum age is below 40 years, 12.3% (Fig 4A). Consequently, when comparing the difference in age structure between HRD scenario and the scenarios considering only one of the two disturbances (i.e., H and RD scenarios), it resulted to be statistically significant (H: χ^2^_(5,N = 12)_ = 8173.6, p< 0.001, RD: χ^2^_(5,N = 12)_ = 906.9, p< 0.001). Values regarding the forest structures at the end of all scenarios are reported in Table E in S1 File.

#### Forest structure and harvesting prescriptions

**Fig 5:**
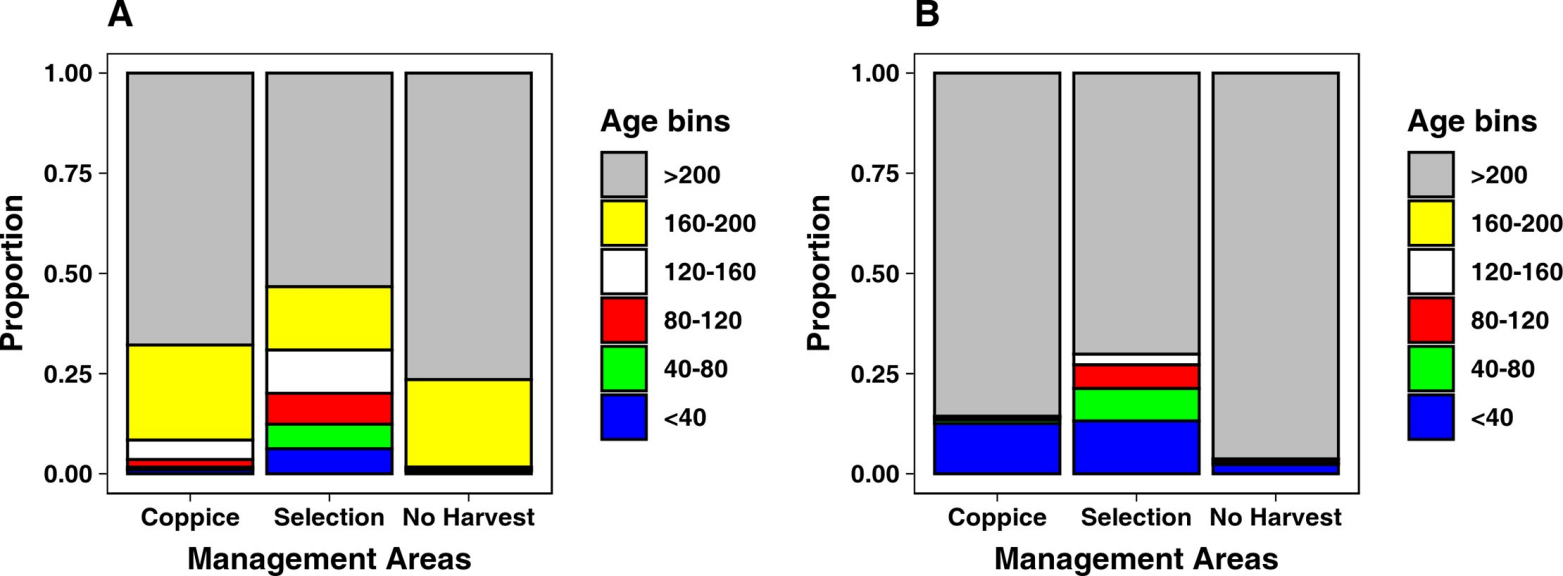
Proportion of age bin distribution between management areas for Harvesting scenario (A), and Harvesting & Roe deer scenario (B).

Due to the application of different prescriptions, different management areas result in different forest structures (Table G in S1 File). In the H scenario, the coppice management area has an age structure shifted towards older ages, while Selection management areas shows a more balanced distribution. In No Harvest management areas, almost all the cells have a maximum age above 160 years (Fig 5A). The differences in age distribution between management areas resulted all to be significant (Coppice *vs* Selection: χ^2^_(5,N = 12)_ = 5132.4, p< 0.001; Coppice *vs* No Harvest: χ^2^_(5,N = 12)_ = 45, p< 0.001; Selection *vs* No Harvest: χ^2^_(5,N = 12)_ = 258.5, p< 0.001). In the HRD scenario, Selection management area resulted to have a higher percentage of young forest, as 27% of cells have a maximum age lower than 120 years. Coppice and No Harvesting management areas have a quite similar age structure, markedly shifted towards older ages, as the >200 year age class includes at least 85% of the cells (Fig 5B). Nevertheless, the chi-squared tests returned the differences as significant, for all the management areas: χ^2^_(4,N = 10)_ = 514.3, p< 0.001 for Coppice against No Harvesting, χ^2^_(4,N = 14)_ = 4902.1, p< 0.001 for Selection against Coppice, and χ^2^_(4,N = 10)_ = 166.4, p< 0.001 for Selection against No Harvesting.

### Species richness

In ND scenario, there is a clear prevalence of cells containing 3 species (40.1%), while the rest of the area is mostly composed in equal parts by cells with 1 or 2 species. It is worth noting the presence of cells containing 4 or 5 different species, although in a small percentage (Table 2). The species richness for the ND scenario is significantly different from one of the other scenarios (H scenario: χ^2^_(3,N = 8)_ = 7669.1, p< 0.001, RD scenario: χ^2^_(3,N = 8)_ = 300.2, p< 0.001, and HRD scenario: χ^2^_(3,N = 8)_ = 6051.3, p< 0.001). The species richness in H scenario is the lowest of all scenarios, with the highest percentage of cells containing a single species (31.3%), and a general shift towards lower values of per-cell species richness (Table 2). Conversely, RD scenario shows the highest per-cell species richness among our simulations, even though there are no cells containing 5 different species (Table 2).

**Table 2:** Percentages of study area coverage, by per-cell species richness and scenario.

Scenarios	1 Spp	2 Spp	3 Spp	4 Spp	5 Spp
No Disturbance	25.6	26.0	40.1	8.1	0.2
Harvesting	31.3	53.2	15.0	0.4	0.0
Harvesting & Roe deer	25.7	54.1	20.0	0.3	0.0
Roe deer	19.7	28.7	44.5	7.1	0.0

Finally, in HRD scenario, the per-cell species richness is shifted towards lower values, with the highest percentage falling in the 2-species class (54.1%). The remaining percentage is almost equally divided between the 1-species and the 3-species classes (Table 2). We compared the distribution of species richness of this scenario against the other scenarios including disturbance sources: it resulted to be significantly different (H scenario χ^2^_(3,N = 8)_ = 346.0, p< 0.001; RD scenario χ^2^_(3,N = 8)_ = 27385.0, p< 0.001).

### Forest loss

**Fig 6:**
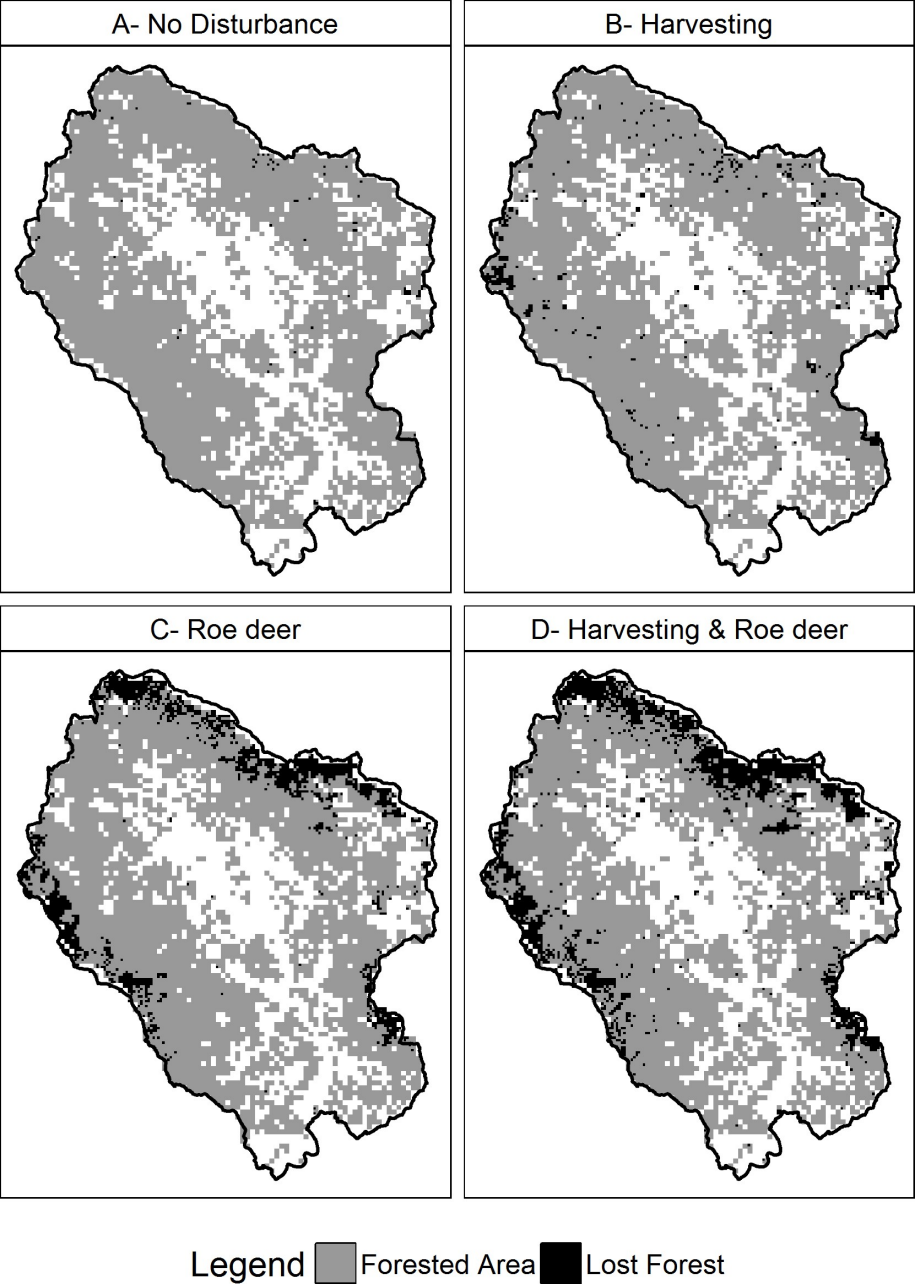
Forest loss areas at the end of simulation in all 4 scenarios.

At the end of the simulations, our forested area showed a reduction in extent in all 4 scenarios. The magnitude of the reduction differs amongst scenarios, from 0.3% of ND scenario to 15.2% of HRD scenario (Table 3). It seems that most of forest loss concentrated along the outermost areas of the study area (Fig 6). Even if harvesting has an effect on forest loss, it seems that roe deer presence markedly affects forest extent. The cells subjected to forest loss were covered, in ND scenario, mostly by the Fagus forest type, with 75.5% and 89.6% coverage for HRD and RD scenario respectively. Conifers and Mix forest types covers respectively 12.3% and 6.4% for HRD scenario, and 5.8% and 2.0% for RD scenario. Oak-Hornbeam and Mix Broadleaves are represented by smaller proportions (Table 4).

**Table 3:** Forest lost at the end of simulation for each scenario, expressed as percentage of initial forested extent.

Scenarios	Forested area loss (percentage)
No Disturbance	0.27%
Harvesting	2.27%
Roe deer	10.74%
Harvesting & Roe deer	15.19%

**Table 4:** Percentage of forest type coverage of the cells that are lost forest on *Harvesting & Roe deer* and *Roe deer* scenario.

Forest types	Harv&Roe	Roe deer
Fagus	75.5%	89.6%
Castanea	0.3%	0%
Oaks	0.1%	0.2%
Oaks-Hornbeam	2.4%	0.6%
Conifers	12.2%	5.8%
Mix Broadleaves	1.9%	0.7%
Mix	6.4%	2.0%
Not Forested	1.3%	1.1%

### Roe deer presence effects

#### Effect on harvest extent

**Fig 7:**
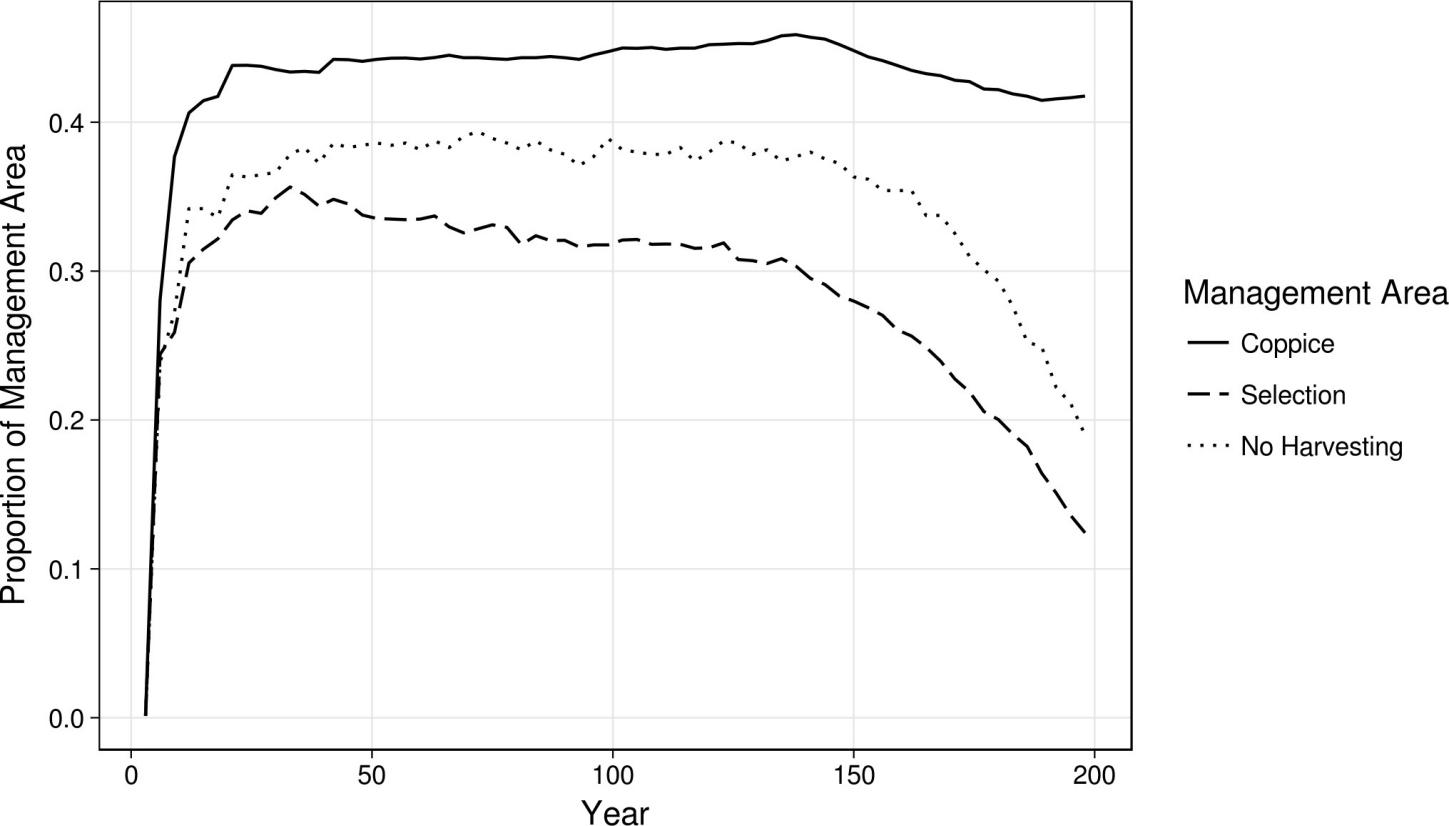
Extent of roe deer impact expressed as proportion of each management area affected for each time step.

We applied the Friedman’s test and its *post-hoc* tests to data from the two scenarios with harvesting, to test for differences in harvesting yield due to the presence of roe deer. The tests have been run for each prescription. Only two tests, for Castanea Coppice and Conifers Reduction prescriptions in Coppice management area, reported significant results (p< 0.001 for both prescriptions). Quercus Coppice prescription in Coppice management area reported results are at the significance limit, p = 0.058. All the other tests run for the other prescriptions, in all management areas, reported non-significant results (Table J in S1 File). To test for differences in roe deer impact amongst management areas, we used the Friedman’s *post-hoc* tests on the HRD scenario data. The impact, expressed as proportion of management area affected, is highest on Coppice management areas, followed by No Harvest and Selection management areas (Fig 7). Those differences are statistically significant, as returned by the *post-hoc* tests: p = 0 for Coppice against Selection, p< 0.001 for both Coppice against No Harvest, and Selection against No Harvest.

#### Effect on species abundance

The analysis of the roe deer effect on species abundance have been conducted comparing the results between ND scenario’s and RD scenario’s data. Five species out of the 12 species considered are not significantly affected by roe deer presence: three of those species are considered not palatable in the simulation (*Pinus nigra*, *Pinus pinaster*, and *Robinia pseudoacacia*), while the other two are considered as palatable, (*Castanea sativa* and *Quercus cerris*). The latter has a p-value on the significance limit: p = 0.058. Most of the palatable species have a decreasing trend in RD scenario: *Abies alba*, *Acer pseudoplatanus*, *Fagus sylvatica*, and *Fraxinus ornus*. Two species show increasing trends (*Ostrya carpinifolia* and *Pseudotsuga menziesii*), while the remaining show a quite stable trend (see Table H in S1 File).

## Discussion

In our study we have examined the effects of two forest disturbances, separately and in combination. These events are difficult to measure empirically, especially if considered on large-scale and long-term. Both the disturbances modelled have an effect on forest development and on all the ecological variables considered, i.e. species abundance, species richness and forest structure. When the two disturbances were combined, their effects interact smoothing or sharpening the outcomes in relation to the ecological aspect considered. Roe deer, at the densities modelled in our simulations, seems to have a positive effect on forest development. Even if it has a negative impact on the abundance of palatable species, it increases species richness, and as it impacts only very young trees, it allows older trees to grow undisturbed. Nonetheless, consuming young trees it opens niches for other trees to colonize, favoring a continuous abundance of young trees, as shown by the high percentage of trees younger than 40 years in the scenarios where roe deer is considered. On the other hand, forest management has a stronger impact on forest development. It decreases species richness, as it favors mono-cultural patches and it shifts the age structure of the forest towards lower values but, in the timespan of our simulations, it returns a multi-layered forest, with an age distribution less clumped on very young and very old trees. When the two disturbances are simultaneously considered, species richness is quite low, driven mostly by forest management, and the age structure is shifted towards lower values, driven mostly by roe deer. This is, in fact, the scenario where the very young trees are most abundant, due to the synergistic effect of the disturbances.

We acknowledge some trade-offs of our approach, as it does not include climate change and considers roe deer density as homogeneous in the whole study area. However, our aim was not to predict how the forest will developed, but to identify how the two disturbances (i.e., harvesting and roe deer) would interact and affect the ecological dynamics considered. To add climate change and variable roe deer density to our simulations would have increased the complexity of the scenarios and would have made it more difficult to disentangle the effects of each disturbance. Moreover, they would not have improved our results, but only made them more complex without necessarily yield an increase in realism.

### Forest types

At the end of the simulation of the ND scenario, conifer-related (Conifers and Mix) and shade-intolerant (Oaks and Castanea) forest types show a marked decrease in abundance. The former is most probably because conifers are not native in the area and seldom reproduce in the second growth forest. They resist to some degree until the end of the simulation because of the longevity of the species, which for almost all species is higher than the simulation’s length. Oaks and Castanea forest types are probably hindered by their low shade tolerance and dispersal capabilities, which don’t allow them to survive under the high shade species and reduce their ability to colonize neighbouring areas. Moreover, *Castanea sativa* is on the edge of its suitable elevation range in this area, and without proper management, the species suffers an inevitable decline. In our simulation, the magnitude of this decline is low because of the great potential longevity of the species (i.e. 600 years) compared to the length of our simulations (i.e. 200 years).

Harvesting, by targeting some species and aiming at maximizing the harvest yield in the long run, causes directly and indirectly a shift in species composition. That happens by both planting the desired species and eradicating the undesired ones, that can compete for resources, and changing the inter-specific competition by deeply modifying the environment. The application of harvesting prescriptions changed the forest types distribution, reducing Conifers and increasing Oaks distribution. Eradication of coniferous alien species is one of the aims of the current management plan, so it was explicitly applied into prescriptions. Oaks increased distribution is due to the management strategy, related to the high commercial value of those species, which in our prescriptions is emphasized by simulating oak sprouting with the prescription that deals with oaks coppice management.

Ungulate impact is shown in the literature to alter species abundance, as preferred (palatable) species are subject to a selection. Our simulation results are in accord with literature findings, as they show a clear decrease of the forest types composed by roe deer preferred species. Two forest types increased during the simulation, Oak-Hornbeam and Mix Deciduous. This increase seems to be linked to a loss of pure oak plantation due to the colonization by other species (especially *Ostrya carpinifolia*) for the former, and to a more general enrichment of the cells’ species pool for the latter. These changes, however, should not be attributed to roe deer impact only, as they are present–although in smaller magnitude–in the ND scenario. This leads to the conclusion that changes in forest types distribution due to roe deer impact are more related to an increased richness in per-cell species rather than a reduction in the abundance of single species across the landscape.

When considering the two disturbances combined, in HRD scenario, we can see the effects of both disturbances on forest types distribution: the increase of Oaks forest type in spite of Oak-Hornbeam, and the strong decrease of Mix and Conifers forest types are due to harvesting disturbance; while the decrease of Fagus and the relatively high value of Oak-Hornbeam are due to roe deer impact. It’s interesting to note that this is the scenario where Castanea abundance has the highest value, and this is probably due to the colonization by *Castanea sativa* of the cells left vacant by *Fagus sylvatica*. This hypothesis is supported by the spatial distribution of Castanea and Fagus at the end of the simulation.

Regarding the effect of roe deer presence on harvesting yield, results show that roe deer impact did reduce the harvested area–as we predicted—but only for the coppice management area. That makes sense, as it is the one with the shortest rotation period, and therefore the one with the highest amount of cells with saplings, which are the most affected by roe deer impact (which targets saplings up to 6 years). The presence of roe deer in the other management areas, where the ages targeted by harvesting prescriptions are older, didn’t cause a significant difference in harvest yield.

### Forest structure

The forest structure of ND scenario indicates, as expected, the presence of a mature forest, as more than 96% of the cells have a maximum age higher than 160 years. This was expected as the only cause of mortality in this scenario is senescence, and the time span of our simulation is shorter than the longevity of the majority of the species considered. When considering the managed forest of H scenario, the age structure will strongly depend on the management practice applied. Our results show a shift of the forest structure towards younger ages, which is due to the selection cutting in the Selection management area. The prescriptions applied in Selection management area targeted older cohorts, increasing the number of cells composed by younger trees. The Coppice management area, on the other hand, shows an age structure where more than 90% of cells host trees older than 160 years. This seems counter intuitive, as a higher percentage of young trees would be expected in coppice stands, but as mentioned before, the software outputs the age of the oldest age cohort in the cell. As the prescriptions in this management area target only young trees, leaving the older ones–inside the same cells- aging without disturbances, the resulting forest structure will be shifted towards older ages.

The forest structure of RD scenario is strongly shifted towards older ages, as 90% of cells have trees older than 200 years. Interestingly, the youngest ages (up to 10 years) are very well represented, about twice the abundance returned in the ND scenario. This gap in forest structure between very young and old ages is explained by the age cohorts targeted by the roe deer impact prescription. As roe deer are targeting saplings up to 6 years old, the cohorts that at the beginning of the simulation were older than that age could grow undisturbed, and as the simulation models a 200 years span, at the end all of those cohorts will be older than 200 years. At the same time, saplings are regularly removed by roe deer, leaving cells at disposal for colonization by other species from the surrounding cells, which, once established, will be potentially targeted by roe deer prescription again. As the roe deer prescription is applied in the whole area at each time step, the presence of saplings will be widespread in the area, but hidden–in our model output–by the presence of older cohorts in the same cells. The percentage of cells that we categorized as younger than 10 years are probably those were the older cohorts died because they reach their longevity limit, as there is no other factor, in this scenario, that would cause the disappearance of older age cohorts.

In HRD scenario, the forest structure is shifted towards older ages as we predicted, but there is a surprisingly high proportion of very young trees. When we look in detail at the proportion of trees younger than 100 years, we can see that this scenario is the one where the proportion of trees younger than 10 years is the highest. The dynamics identified in RD scenario, where roe deer impact was “clearing” all the saplings from a cell, leaving it open for establishment, are reinforced here on a wider spatial scale by the Coppice prescriptions, which are targeting age cohorts unreachable by roe deer and planting oaks saplings instead, giving Roe deer prescription a wider area to be, potentially, applied.

### Species richness

Species richness in ND scenario increased at the end of simulation–see Figure K in S1 File -, indicating a shift towards a landscape composed of mixed deciduous species assemblages. Forestry practices in the past introduced new alien species in the area, and devoted wide areas to the cultivation of single economic important species, creating a landscape composed of mono-cultural patches. Once the human factor is removed, the species with higher shade tolerance values tend to colonize those patches, increasing the average per-cell species richness.

As species richness is affected by harvesting, as it shows a shift towards lower values in H scenario. The presence of openings in the forest, as the ones created by some forest management practice, usually favors the establishment of species different from the ones present in the canopy, locally increasing species richness [76,77]. This does not happen in our simulations for two reasons: first, as mentioned before, planting–that occurs after coppicing prescriptions- prevents the establishment of other species in that cell; second, in LANDIS-II framework the cell is the smaller entity, and is considered homogeneous. In other words, openings smaller than the cell size do not exist in the simulation.

RD scenario resulted to have the highest values of species richness, as suggested by several authors for ungulates [17,78]. Moreover, if compared with ND scenario, the increase in species richness appears around the end of the simulation–see Figure K in S1 File -, suggesting that the magnitude of the phenomenon may have an increasing trend that goes beyond the end of the simulation. However, species richness seems to be influenced more by harvesting than by roe deer impact: in HRD scenario the distribution of per-cell species number resulted to be shifted towards lower values, resembling the distribution obtained when only harvesting was considered. The influence of roe deer impact can be seen in the light shift towards higher values with respect to the species richness distribution of the H scenario, but this effect is not enough to shift the distribution closer to the ND’s or RD scenario’s ones.

### Forest loss

Our results show the loss of some areas of forest in all 4 of our scenarios, but the extent of this loss shows quite a wide range. It seems apparent that roe deer impact has a major effect on this phenomenon, as the scenarios considering roe deer are the ones that experienced the loss to the widest extent. Harvesting has a certain effect too, as when considered alone it returns a forest loss which is several times the one returned by ND scenario. The two disturbances, when considered combined, have an additive effect, which leads to the loss of wide areas of forest, mostly along the edges of the study area. The fact that the lost areas are located mostly along the edges suggests that the phenomenon is probably biased by a modelling artifact, which is the map edge effect, artificially limiting colonization sources into border cells. If that bias were to be removed, the outer cells of our study area wouldn’t be “isolated”, and maybe the extent of forest loss would be smaller. The location of these map edge cells would explain why the majority of those cells would have been hosting Fagus forest type. This is probably due to the location of this forest type, which finds its ecological niche at higher elevations that are mostly along the borders of the study area. Moreover, this forest type is targeted by both disturbances. In addition, the high shade-tolerance value of this species prevents both other species to grow below its canopy, and its colonization of vacant cell. So when Fagus is eradicated from these cells, they remain empty, as they are surrounded by either other Fagus cells or the artificial edge. On the other hand, results show that several areas where forest was lost are far from the edges of the study area, indicating that the forest loss might be an actual effect of the disturbances, despite the bias concerning the study area borders.

Forest loss does not currently raise concerns in our study area, where agricultural areas extent is decreasing, leaving space for spontaneous reforestation. Nonetheless, it would be interesting to further investigate the causes of this phenomenon, in case the trend of agricultural areas would reverse its course.

Our analyses were focused on roe deer because the species is ubiquitous in our study area, and forest managers consider it to have a negative impact both on forest development and on harvesting yield. Our results show not only that roe deer has not a negative impact on forest development, but it seems to have a positive effect. It increases the overall species richness and shift the forest structure towards older ages. Although it has a negative impact on harvesting yields, it results overall not to be significant. Nonetheless, these seemingly positive effects are counterbalanced by the marked effect roe deer impact has on forest loss, as scenarios considering roe deer have a forest loss extent 5 times greater than the other scenarios. While roe deer impact alone does not seems to be a hazard for forest development, when it is associate with forest management, the synergistic effect of the two disturbance cause a combination of loss in both species richness and forest extent which might be of concern for future forest functionality. A forest with few species and a tendency for forest loss might results dependent on forest management to survive in the long run.

## Conclusions

Working with models always implies simplifications and generalizations [79]. Our simulations provided some insight on the development of the forest under the effects of the two disturbances considered; nonetheless they should be viewed as indications of the resulting trends rather than actual future predictions. Our results show that the two disturbances are interacting in all aspects of forest ecology we considered. Forest types distribution and forest structure are affected by both disturbances, but it does not seem that one disturbance has a stronger influence on those aspects. On the other hand, the species richness seems to be strongly driven by harvesting. The deforestation depicted in the results should be look at with caution. Even if the trend seems to be present in all four scenarios considered, model constraints and simplifications could have had a strong effect on this aspect of forest ecology. The combination of the two disturbances, with the magnitude simulated in the modelling, does not seem to be a hazard for the forest development in our study area. None of the ecological parameters examined, compared to the simulation without disturbances, showed alterations that raised concerns. In our simulations, the presence of an ungulate, which forest managers suggest are causing a marked loss in revenues due to browsing impact, does not seems to significantly affect harvest yield, and apparently increases biodiversity. This suggests that, with the forest management and ungulate impact levels simulated in our modelling, the effects of ungulate presence are overall positive based on this metric. Still, it’s important to remember that only one out of four of the ungulate species present in the area has been considered in the model, and their combined effect could have markedly modified the outcome of the modelling. This “missing part” in our modelled scenarios is due to a lack of data about the impact of those species on the forest in our area, as the software would have accommodated it. In fact, despite the constraints imposed by the modelling effort, we found LANDIS-II to be a very flexible tool, which allows simulating a wide variety of ecological situations at different–user selected- levels of complexity. The ability to show the trends the landscape will face under different situation is very useful for virtually testing managing options and different scenarios, a practice that is impossible or impractical to apply empirically. Moreover, the results of the simulations are quite rich in details, enabling scientists or managers from other disciplines to use those results as a base for their analysis (e.g. [80]). Managers can use LANDIS-II to simulate their disturbance(s) regime and analyze the emerging ecological trends, to see if these go towards their management goals. If that is not the case, they can modify disturbance regimes in the simulations to identify which management strategy would lead them towards their goals. Moreover, if a new disturbance appears in the area (e.g. ungulate population, wind throws, fires, etc.) they can add it to their simulations and see how this will affect the resulting trends.

Although the results in our paper can be generalized only to areas with similar initial conditions, the result from our simulations can be taken as indications of which trends will emerge when one or both disturbances considered are present in a certain area.

## Supporting information

S1 File Graphic and table supporting the results of the paper. **Table A Life traits parameters for the selected species.** Shown values are: Longevity (years), Sexual Maturity age (years), Shade Tolerance (ranked increasingly from 1 to 5), Fire Tolerance (ranked increasingly from 1 to 5), Effective and Maximum seed dispersal distance (meters), Probability of Vegetative Reproduction, Minimum and Maximum re-sprout age (years), type of Post Fire Regeneration (Seronoty, Resprout or None). **Table B Establishment probabilities of each species for the 5 ecoregions considered. Table C List of prescriptions applied in Harvesting and Harvesting & Roe Deer scenarios. Table D Percentages of study area coverage at the end of simulations, by major forest type and scenario. Table E Percentages of study area coverage.** By age bins and scenario, for all age classes (top), and for cells with maximum age lower than 100 years (bottom). **Table F Table of p-values of Friedman’s post-hoc tests for the comparison of forest types abundance between different scenarios.** p-values in italic font are not significant. **Table G Percentages of study area coverage.** By Management Areas’ age structure, relative to Harvesting scenario (top), and Harvesting & Roe deer scenario (bottom). **Table H Table of trends for each species, and p-values for the significance of the difference of abundance between the two scenarios.** Minus sign (“-”) indicates that there are no differences to test. **Table I Physiological parameters used by PnET-II for LANDIS-II. Table J Table of p values of Friedman’s post-hoc tests for the comparison of harvesting yields.** For each prescription between Harvesting and Harvesting & Roe deer scenarios. p-values in italic font are not significant. Minus sign (“-”) indicates that there are no differences to test. **Figure K Species richness distribution for each scenario at time intervals of 20 years.**
(DOCX)

S2 File Input files for running LANDIS-II simulations. (DOCX)

S3 File Initial community data set for LANDIS-II. (TXT)

S4 File Initial community map for LANDIS-II. (TIF)

S5 File Ecoregions map for LANDIS-II. (TIF)

S6 File Management areas map for LANDIS-II. (TIF)

S7 File Stand map for LANDIS-II. (TIF)
