# Supplementary material for: Effects of forest management and roe deer impact on a mountain forest development in the Italian Apennines: A modelling approach using LANDIS-II
Source: PLoS One. 2019 Nov 6;14(11):e0224788. doi: 10.1371/journal.pone.0224788 (PMC6834274; doi:10.1371/journal.pone.0224788)

# Appendix

**Table A** Life traits parameters for the selected species. Shown values are: Longevity (years), Sexual Maturity age (years), Shade Tolerance (ranked increasingly from 1 to 5), Fire Tolerance (ranked increasingly from 1 to 5), Effective and Maximum seed dispersal distance (meters), Probability of Vegetative Reproduction, Minimum and Maximum re-sprout age (years), type of Post Fire Regeneration (Seronoty, Resprout or None).

| Species | Longevity | Sexual Maturity | Shade Tol. | Fire Tol. | Seed dispersal distance | | Vegetative reprod. prob. | Min sprout age | Max sprout age | Post-fire Regen. |
| --- | --- | --- | --- | --- | --- | --- | --- | --- | --- | --- |
|  |  |  |  |  | Effective | Max. |  |  |  |  |
| Abies | 300 | 70 | 4 | 3 | 50 | 160 | 0 | 0 | 0 | None |
| Acer | 300 | 15 | 4 | 2 | 100 | 300 | 0.8 | 1 | 100 | Resprout |
| Castanea | 600 | 15 | 3 | 2 | 20 | 30 | 0.9 | 1 | 600 | Resprout |
| Fagus | 300 | 60 | 5 | 2 | 25 | 50 | 0.75 | 1 | 100 | Resprout |
| Fraxinus | 200 | 20 | 3 | 2 | 50 | 140 | 0.8 | 1 | 80 | Resprout |
| Ostrya | 150 | 30 | 4 | 2 | 80 | 180 | 0.8 | 1 | 100 | Resprout |
| Pinus_nig | 300 | 30 | 2 | 3 | 40 | 100 | 0 | 0 | 0 | None |
| Pinus_pin | 200 | 15 | 1 | 4 | 40 | 300 | 0 | 0 | 0 | None |
| Pseudotsuga | 700 | 20 | 3 | 5 | 100 | 250 | 0 | 0 | 0 | None |
| Quercus_cer | 350 | 40 | 2 | 3 | 20 | 40 | 0.9 | 1 | 80 | Resprout |
| Quercus_pub | 350 | 50 | 2 | 3 | 20 | 40 | 0.75 | 1 | 80 | Resprout |
| Robinia | 100 | 15 | 2 | 1 | 30 | 120 | 0.9 | 1 | 100 | Resprout |

**Table B** Establishment probabilities of each species for the 5 ecoregions considered.

|  | Ecoregions | | | | |
| --- | --- | --- | --- | --- | --- |
| Species | 1 | 2 | 3 | 4 | 5 |
| *Abies alba* | 0.5 | 0.7 | 0.5 | 0.3 | 0.1 |
| *Acer pseudoplatanus* | 0.5 | 0.7 | 0.7 | 0.4 | 0.2 |
| *Castanea sativa* | 0.1 | 0.6 | 0.6 | 0.2 | 0.1 |
| *Fagus sylvatica* | 0.9 | 0.7 | 0.5 | 0.2 | 0.1 |
| *Fraxinus ornus* | 0.1 | 0.4 | 0.7 | 0.7 | 0.6 |
| *Ostrya carpinifolia* | 0.1 | 0.7 | 0.7 | 0.7 | 0.7 |
| *Pinus nigra* | 0.1 | 0.4 | 0.6 | 0.4 | 0.1 |
| *Pinus pinaster* | 0.1 | 0.2 | 0.3 | 0.4 | 0.6 |
| *Pseudotsuga menziesii* | 0.1 | 0.7 | 0.6 | 0.2 | 0.1 |
| *Quercus cerris* | 0.1 | 0.8 | 0.7 | 0.4 | 0.1 |
| *Quercus pubescens* | 0.1 | 0.2 | 0.4 | 0.6 | 0.9 |
| *Robinia pseudoacacia* | 0.1 | 0.7 | 0.7 | 0.7 | 0.7 |

**Table C** List of prescriptions applied in *Harvesting* and *Harvesting & Roe Deer* scenarios.

| Prescription | Target species | Target age cohorts | Management area | Target area (%) |
| --- | --- | --- | --- | --- |
| Quercus Coppice (planting oaks) | *Quercus cerris*  *Quercus pubescens* | 20-40  20-40 | Coppice | 4.3% |
| Castanea coppice | *Castanea sativa* | 20-40 | Coppice | 1.7% |
| Broadleaves coppice | *Ostrya carpinifolia*  *Fagus sylvatica* | 20-40  20-40 | Coppice | 1.3% |
| Conifers reduction | *Abies alba*  *Pinus nigra*  *Pseudotsuga menziesii* | All  All  All | Coppice | 5% |
| Thinning | *Castanea sativa*  *Fagus sylvatica*  *Ostrya carpinifolia*  *Quercus cerris*  *Quercus pubescens*  *Pseudotsuga menziesii* | 40-50  40-90  40-60  40-80  40-90  All | Selection | 1% |
| Selection cutting | *Castanea sativa*  *Fagus sylvatica*  *Ostrya carpinifolia*  *Quercus cerris*  *Quercus pubescens*  *Pseudotsuga menziesii* | 50-100  90-140  60-150  80-200  90-200  All | Selection | 1% |
| Conifers eradication | *Abies alba*  *Pinus nigra*  *Pseudotsuga menziesii* | All  All  All | Selection | 0.9% |
| Roe deer | *Castanea sativa*  *Fagus sylvatica*  *Quercus cerris*  *Quercus pubescens*  *Abies alba*  *Acer pseudoplatanus*  *Fraxinus ornus* | 1-3  1-3  1-6  1-6  1-6  1-6  1-6 | All Management Areas | 100% |

**Table D** Percentages of study area coverage at the end of simulations, by major forest type and scenario.

| Scenarios | Fagus | Castanea | Oaks | Oak-Hornbeam | Conifers | Mix-Broadleaves | Mix |
| --- | --- | --- | --- | --- | --- | --- | --- |
| No Disturbance | 16.9 | 2.1 | 1.3 | 37.8 | 2.8 | 19.4 | 19.5 |
| Harvesting | 18.9 | 4.8 | 34.7 | 11.7 | 1.5 | 25.6 | 2.2 |
| Roe deer | 4.6 | 3.6 | 1.4 | 45.1 | 4.6 | 23.9 | 16.6 |
| Harvesting &  Roe deer | 6.0 | 6.9 | 32.1 | 25.9 | 0.5 | 27.6 | 0.7 |

**Table E** Percentages of study area coverage, by age bins and scenario, for all age classes (top), and for cells with maximum age lower than 100 years (bottom).

| All age classes | | | | | | |
| --- | --- | --- | --- | --- | --- | --- |
| Scenarios | <40 | 40-80 | 80-120 | 120-160 | 160-200 | >200 |
| No Disturbance | 1.6 | 1.3 | 2.2 | 0.8 | 13.3 | 80.8 |
| Harvesting | 2.8 | 2.6 | 3.9 | 6.7 | 20.9 | 63.2 |
| Roe deer | 4.8 | 1.4 | 2.6 | 0.7 | 0.1 | 90.4 |
| Harvesting & Roe deer | 12.3 | 3.0 | 2.2 | 1.0 | 0.0 | 81.4 |
| Age classes < 100 years old | | | | | | |
| Scenarios | <10 | 10-20 | 20-40 | 40-60 | 60-80 | >80 |
| No Disturbance | 14.6 | 9.3 | 18.6 | 20.4 | 14.5 | 22.7 |
| Harvesting | 11.2 | 11.7 | 22.0 | 28.8 | 12.6 | 13.7 |
| Roe deer | 37.0 | 10.2 | 18.0 | 12.6 | 6.8 | 15.4 |
| Harvesting & Roe deer | 53.2 | 6.9 | 13.7 | 12.4 | 5.8 | 8.0 |

**Table F** Table of p-values of Friedman’s *post-hoc* tests for the comparison of forest types abundance between different scenarios. p-values in italic font are not significant.

| **Fagus** | *No Disturbance* | *Harvesting* | *Harvesting & Roe deer* |
| --- | --- | --- | --- |
| *Harvesting* | <0.001 |  |  |
| *Harvesting & Roe deer* | 0.043 | <0.01 |  |
| *Roe deer* | <0.01 | <0.001 | <0.001 |
| **Castanea** | *No Disturbance* | *Harvesting* | *Harvesting & Roe deer* |
| *Harvesting* | <0.001 |  |  |
| *Harvesting & Roe deer* | <0.001 | *0.95* |  |
| *Roe deer* | <0.01 | <0.001 | <0.001 |
| **Oaks** | *No Disturbance* | *Harvesting* | *Harvesting & Roe deer* |
| *Harvesting* | <0.001 |  |  |
| *Harvesting & Roe deer* | <0.001 | *0.11* |  |
| *Roe deer* | *0.73* | <0.001 | <0.001 |
| **OakHornbeam** | *No Disturbance* | *Harvesting* | *Harvesting & Roe deer* |
| *Harvesting* | <0.001 |  |  |
| *Harvesting & Roe deer* | <0.001 | <0.01 |  |
| *Roe deer* | 0.036 | <0.001 | <0.001 |
| **Conifers** | *No Disturbance* | *Harvesting* | *Harvesting & Roe deer* |
| *Harvesting* | <0.001 |  |  |
| *Harvesting & Roe deer* | <0.001 | 0.028 |  |
| *Roe deer* | *1.0* | <0.001 | <0.001 |
| **MixBroadleaves** | *No Disturbance* | *Harvesting* | *Harvesting & Roe deer* |
| *Harvesting* | <0.001 |  |  |
| *Harvesting & Roe deer* | <0.001 | <0.01 |  |
| *Roe deer* | <0.01 | <0.001 | <0.01 |
| **Mix** | *No Disturbance* | *Harvesting* | *Harvesting & Roe deer* |
| *Harvesting* | <0.001 |  |  |
| *Harvesting & Roe deer* | <0.001 | <0.01 |  |
| *Roe deer* | <0.01 | <0.01 | <0.001 |

**Table G** Percentages of study area coverage, by Management Areas’ age structure, relative to *Harvesting* scenario (top), and *Harvesting & Roe deer* scenario (bottom).

| *Harvesting* scenario | | | | | | |
| --- | --- | --- | --- | --- | --- | --- |
| Management Area | <40 | 40-80 | 80-120 | 120-160 | 160-200 | >200 |
| Coppice | 1.0 | 0.7 | 1.9 | 4.9 | 23.7 | 67.8 |
| Selection | 6.3 | 6.1 | 7.7 | 10.8 | 15.8 | 53.3 |
| No Harvesting | 0.6 | 0.3 | 0.5 | 0.3 | 21.8 | 76.5 |
| *Harvesting & Roe deer* scenario | | | | | | |
| Management Area | <40 | 40-80 | 80-120 | 120-160 | 160-200 | >200 |
| Coppice | 12.6 | 0.9 | 0.5 | 0.4 | 0.0 | 85.6 |
| Selection | 13.2 | 8.1 | 5.9 | 2.6 | 0.0 | 70.1 |
| No Harvesting | 2.4 | 0.4 | 0.8 | 0.2 | 0.0 | 96.3 |

**Table H** Table of trends for each species, and p-values for the significance of the difference of abundance between the two scenarios. Minus sign (“-”) indicates that there are no differences to test.

| Species | *P*-values | Trend (No Disturbance *vs* Roe deer) |
| --- | --- | --- |
| *Abies alba* | 0.002 | Decreasing |
| *Acer pseudoplatanus* | 0.002 | Decreasing |
| *Castanea sativa* | - | Stable |
| *Fagus sylvatica* | 0.005 | Decreasing |
| *Fraxinus ornus* | 0.002 | Decreasing |
| *Ostrya carpinifolia* | 0.002 | Increasing |
| *Pinus nigra* | 0.48 | Stable |
| *Pinus pinaster* | 0.65 | Stable |
| *Pseudotsuga menziesii* | 0.011 | Increasing |
| *Quercus cerris* | 0.058 | Stable |
| *Quercus pubescens* | 0.025 | Stable |
| *Robinia pseudoacacia* | 1 | Stable |

**Table I** physiological parameters used by PnET-II for LANDIS-II

| Variable | Description | Unit |
| --- | --- | --- |
| AmaxA | Intercept of relationship between foliar N and maximum photosynthetic rate |  |
| AmaxB | Slope of Amax vs N relationship | umol CO_2_/g leaf/s |
| HalfSat | Half saturation light intensity | umol/m^2^/s |
| BFolResp | Respiration as a fraction of maximum photosynthesis |  |
| RespQ10 | Q10 value for foliar respiration |  |
| PsnTMin | Minimum temperature for photosynthesis | °C |
| PsnTOpt | Maximum temperature for photosynthesis | °C |
| AmxFrac | Daily Amax as a fraction of early morning instantaneous rate |  |
| FolRet | Maximum relative growth rate for foliage | % per year |
| SLWmax | Species leaf weight at top canopy | g/m^2^ |
| SLWdel | Change in SLW with increasing foliar mass above | G m^-2^ g^-1^ |
| GDDFolS | Growing degrees days (GDD) at which foliar production begins |  |
| GDDFolE | GDD at which foliar production ends |  |
| GDDWoodS | GDD at which wood production begins |  |
| GDDWoodE | GDD at which wood production ends |  |
| SenescStart | Day of year after which leaf drop can occur |  |
| FolMsMx | Site specific maximum summer foliage biomass | g/m^2^ |
| FolMsMn | Site specific minimum winter foliage biomass | g/m^2^ |
| k | Canopy light extinction constant |  |
| FolNCon | Foliar nitrogen | % |
| FolRelGMax | Maximum relative growth rate for foliage | % per year |
| CFracB | Carbon as a fraction of tissue mass |  |
| RootAlA | Intercept of relationship between foliar and root allocation |  |
| RootAlB | Slope of relationship between foliar and root allocation |  |
| GRspFrac | Growth respiration as a fraction of allocated carbon |  |
| WdMRespA | Wood mantainance respiration as a fraction of gross photosynthesis |  |
| RootMRFPCReserv | Ratio of fine root maintenance respiration to fine root biomass production |  |
| MinWoodFol | Minimum ratio of carbon allocation to wood and foliage |  |
| DVPD1 | Coefficient for photosynthesis reduction due to vapor pressure deficit (VPD) | kPa^-1^ |
| DVPD2 | (VDP) in the power function DVPD = DVPD1 × VPDDVPD2 |  |
| WUECnst | Coefficient in equation for water-use efficiency (WUE) as a function of VPD |  |
| PrecIntF | Fraction of precipitation intercepted and evaporated |  |
| FFlowFr | Set as 0.1 |  |
| FLPctN | Min. N Concentration in foliar litter | % |
| RLPctN | Min. N Concentration in root litter | % |
| WLPctN | Min. N Concentration in wood litter | % |
| FolNConR | Max. fractional increase in N concentrations | % |
| FolNRet | Set at 0.5 |  |
| MaxNStore | Max. N content in PlantN pool | g/m^2^ |
| WoodTrn | Fractional mortality of live wood per year |  |
| RtTrnA | Coefficients for fine root turnover as a function of annual net N mineralization | fraction/year |
| RtTrnB | Coefficients for fine root turnover as a function of annual net N mineralization | fraction/year |
| RootTrnC | Coefficients for fine root turnover as a function of annual net N mineralization | fraction/year |
| WdLitLs | Fractional loss of mass as CO 2 in wood decomposition |  |
| WdCLoss Kho | Decomposition constant for SOM pool (year -1 ) |  |
| NImmobA | Coefficients for fraction of mineralized N reimmobilized as a function of SOM C:N |  |
| NImmobB | Coefficients for fraction of mineralized N reimmobilized as a function of SOM C:N |  |
| SoilRespA | Intercept of relationship between mean monthly temperature and soil respiration | G C /m2/mo |
| SoilRespB | Slope of relationship between mean monthly temperature and soil respiration |  |
| SoilMoistFact | Saturation ratio of the soil |  |
| GDDMin | Based on LINKAGE model which have different baseline T (5.56 degree) from GddFolE GDDWoodS GDDWoodE (0 degree) |  |
| GDDMax | Based on LINKAGE model which have different baseline T (5.56 degree) from GddFolE GDDWoodS GDDWoodE (0 degree) |  |
| CO 2 EFFECT | CO 2 EFFECT on Stomata conductance - True or false |  |

**Table J** Table of p values of Friedman’s post-hoc tests for the comparison of harvesting yields for each prescription between Harvesting and Harvesting & Roe deer scenarios. p-values in italic font are not significant. Minus sign (“-”) indicates that there are no differences to test.

| Management Area | Prescriptions | Friedman’s test *p*-value |
| --- | --- | --- |
| Coppice Management Area | BroadleavesCoppice | - |
|  | CastaneaCoppice | p< 0.001 |
|  | ConifersReduction | p< 0.001 |
|  | QuercusCoppice | *0.058* |
| Selection Management Area | ConifersEradication | *0.083* |
|  | SelectCut | - |
|  | ThinCut | - |

**Figure K** Species richness distribution for each scenario at time intervals of 20 years.


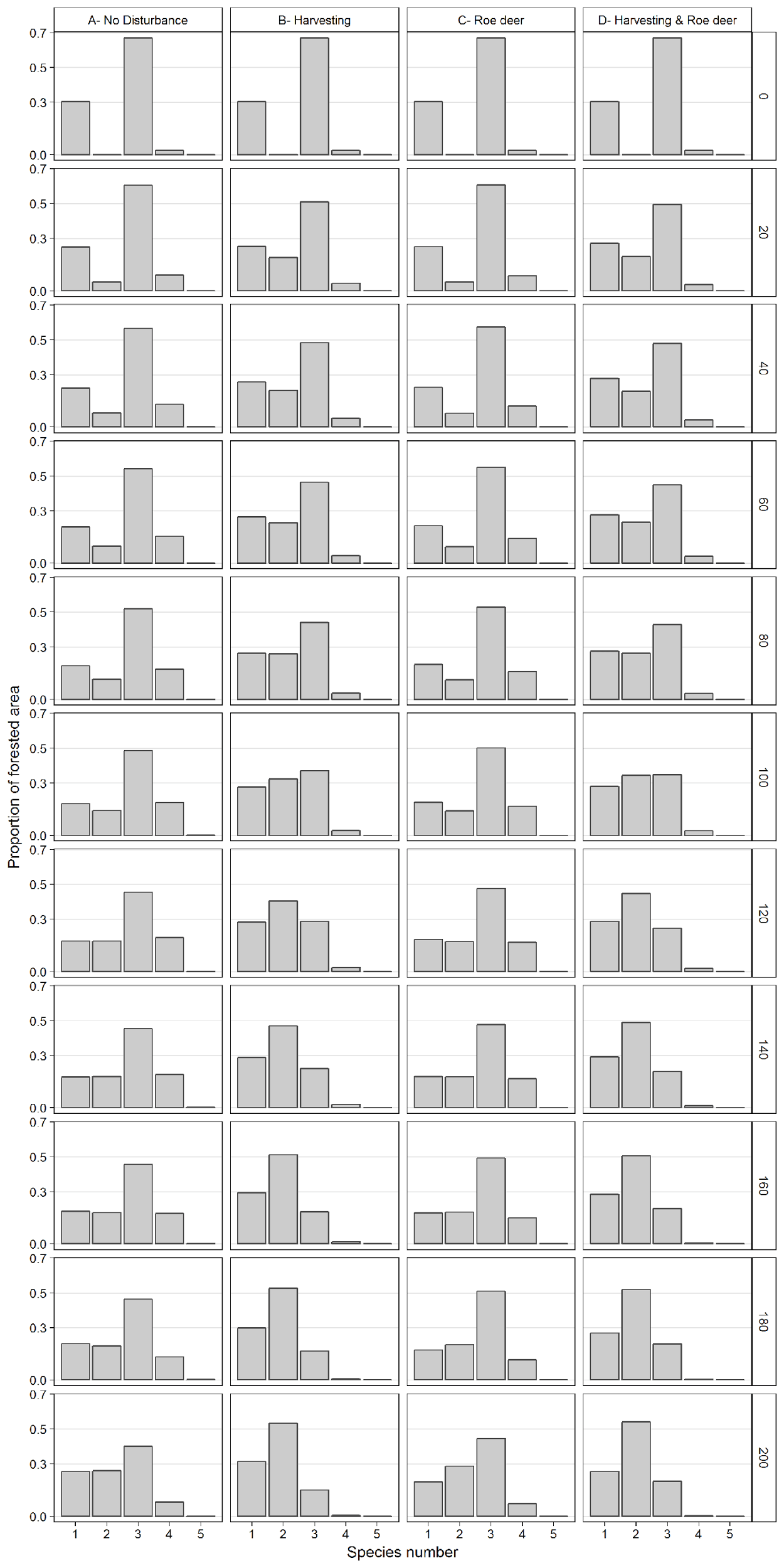

Supplement: S1 File — Table A Life traits parameters for the selected species. Shown values are: Longevity (years), Sexual Maturity age (years), Shade Tolerance (ranked increasingly from 1 to 5), Fire Tolerance (ranked increasingly from 1 to 5), Effective and Maximum seed dispersal distance (meters), Probability of Vegetative Reproduction, Minimum and Maximum re-sprout age (years), type of Post Fire Regeneration (Seronoty, Resprout or None). Table B Establishment probabilities of each species for the 5 ecoregions considered. Table C List of prescriptions applied in Harvesting and Harvesting & Roe Deer scenarios. Table D Percentages of study area coverage at the end of simulations, by major forest type and scenario. Table E Percentages of study area coverage. By age bins and scenario, for all age classes (top), and for cells with maximum age lower than 100 years (bottom). Table F Table of p-values of Friedman’s post-hoc tests for the comparison of forest types abundance between different scenarios. p-values in italic font are not significant. Table G Percentages of study area coverage. By Management Areas’ age structure, relative to Harvesting scenario (top), and Harvesting & Roe deer scenario (bottom). Table H Table of trends for each species, and p-values for the significance of the difference of abundance between the two scenarios. Minus sign (“-”) indicates that there are no differences to test. Table I Physiological parameters used by PnET-II for LANDIS-II. Table J Table of p values of Friedman’s post-hoc tests for the comparison of harvesting yields. For each prescription between Harvesting and Harvesting & Roe deer scenarios. p-values in italic font are not significant. Minus sign (“-”) indicates that there are no differences to test. Figure K Species richness distribution for each scenario at time intervals of 20 years. (DOCX) [file pone.0224788.s001.docx]
