# Supplementary material for: Effects of forest management and roe deer impact on a mountain forest development in the Italian Apennines: A modelling approach using LANDIS-II
Source: PLoS One. 2019 Nov 6;14(11):e0224788. doi: 10.1371/journal.pone.0224788 (PMC6834274; doi:10.1371/journal.pone.0224788)
Supplement: S2 File — (DOCX) [file pone.0224788.s002.docx]

INPUT FILES FOR LANDIS-II

This file contains the input data for the scenario considering both disturbances. To reproduce the other scenarios it is necessary to modify the script in order to de-activate the unwanted extension.

# LANDIS-II input file

LandisData "Scenario"

Duration 200

Species "species.txt"

Ecoregions "ecoregions.txt"

EcoregionsMap "Ecoregions_map_2014.tif"

CellLength 200 << meters

>> Succession Extension Initialization File

>> ---------------------- -------------------

"Age-only Succession" "age-only-succession.txt"

>> Disturbance Extensions Initialization File

>> ---------------------- -------------------

"Base Harvest" "Base-harvest_v2.2.txt"

>> DisturbancesRandomOrder yes << optional parameter; default = no

>> Other Extensions Initialization File

>> ---------------------- -------------------

"Output Max Species Age" "max-spp-age.output.txt"

"Output Age Reclass" "output-age-reclass.txt"

"Output Cohort Statistics" "output-cohort-statistics.txt"

RandomNumberSeed 4357 << optional parameter; default = the seed is randomly generated using the current time

# Age-only succession input file

LandisData "Age-only Succession"

Timestep 3

SeedingAlgorithm WardSeedDispersal

>> Also NoDispersal or UniversalDispersal

InitialCommunities "initial-communities.txt"

InitialCommunitiesMap "InitialCommunitiesMap2014ToExpOK.tif"

DynamicInputFile age-only-succession-dynamic-inputs.txt

# Age-only succession dynamic inputs

LandisData "Dynamic Input Data"

>> Year Ecoregion Species ProbEst

>> -------- -------------

0 eco1 Abies 0.5

0 eco2 Abies 0.7

0 eco3 Abies 0.5

0 eco4 Abies 0.3

0 eco5 Abies 0.1

0 eco1 Acer 0.5

0 eco2 Acer 0.7

0 eco3 Acer 0.7

0 eco4 Acer 0.4

0 eco5 Acer 0.2

0 eco1 Castanea 0.1

0 eco2 Castanea 0.6

0 eco3 Castanea 0.6

0 eco4 Castanea 0.2

0 eco5 Castanea 0.1

0 eco1 Fagus 0.9

0 eco2 Fagus 0.7

0 eco3 Fagus 0.5

0 eco4 Fagus 0.2

0 eco5 Fagus 0.1

0 eco1 Fraxinus 0.1

0 eco2 Fraxinus 0.4

0 eco3 Fraxinus 0.7

0 eco4 Fraxinus 0.7

0 eco5 Fraxinus 0.6

0 eco1 Ostrya 0.1

0 eco2 Ostrya 0.7

0 eco3 Ostrya 0.7

0 eco4 Ostrya 0.7

0 eco5 Ostrya 0.7

0 eco1 Pinus_nig 0.1

0 eco2 Pinus_nig 0.4

0 eco3 Pinus_nig 0.6

0 eco4 Pinus_nig 0.4

0 eco5 Pinus_nig 0.1

0 eco1 Pinus_pin 0.1

0 eco2 Pinus_pin 0.2

0 eco3 Pinus_pin 0.3

0 eco4 Pinus_pin 0.4

0 eco5 Pinus_pin 0.6

0 eco1 Pseudotsuga 0.1

0 eco2 Pseudotsuga 0.7

0 eco3 Pseudotsuga 0.6

0 eco4 Pseudotsuga 0.2

0 eco5 Pseudotsuga 0.1

0 eco1 Quercus_cer 0.1

0 eco2 Quercus_cer 0.8

0 eco3 Quercus_cer 0.7

0 eco4 Quercus_cer 0.4

0 eco5 Quercus_cer 0.1

0 eco1 Quercus_pub 0.1

0 eco2 Quercus_pub 0.2

0 eco3 Quercus_pub 0.4

0 eco4 Quercus_pub 0.6

0 eco5 Quercus_pub 0.9

0 eco1 Robinia 0.1

0 eco2 Robinia 0.7

0 eco3 Robinia 0.7

0 eco4 Robinia 0.7

0 eco5 Robinia 0.7

# Ecoregions

LandisData Ecoregions

>> Map

>> Active Code Name Description

>> ------ ---- ----- -----------

yes 1 eco1 ""

yes 2 eco2 ""

yes 3 eco3 ""

yes 4 eco4 ""

yes 5 eco5 ""

- 0 NotForested "Not forested cells"

- 255 OutStudy "Not study area"

# Base-Harvest v2.2

LandisData "Base Harvest"

Timestep 3

ManagementAreas "./ManagementAreasMap_3areas.tif"

Stands "./StandMap.tif"

>> -----------------------------------------

>> PRESCRIPTIONS

>> PRESCRIPTION FOR "COPPICE" MANAGEMENT AREA

Prescription QuercusCoppice

StandRanking Economic

>> Species Economic Rank Minimum Age

>> ------- ------------- -----------

Quercus_cer 100 20

Quercus_pub 100 20

>> StandAdjacency 20

>> AdjacencyType StandAge

>> AdjacencyNeighborSetAside 20

SiteSelection Complete

CohortsRemoved SpeciesList

>> Species Cohorts removed

>> ------- ---------

Quercus_cer 1-6 20-40

Quercus_pub 1-6 20-40

Castanea 1-3

Fagus 1-3

Abies 1-6

Acer 1-6

Fraxinus 1-6

Plant Quercus_cer Quercus_pub

Prescription CastaneaCoppice

StandRanking Economic

>> Species Economic Rank Minimum Age

>> ------- ------------- -----------

Castanea 100 20

>> StandAdjacency 20

>> AdjacencyType StandAge

>> AdjacencyNeighborSetAside 20

SiteSelection Complete

CohortsRemoved SpeciesList

>> Species Cohorts removed

>> ------- ---------

Castanea 1-3 20-40

Fagus 1-3

Quercus_cer 1-6

Quercus_pub 1-6

Abies 1-6

Acer 1-6

Fraxinus 1-6

>> Plant Castanea

Prescription BroadleavesCoppice

StandRanking Economic

>> Species Economic Rank Minimum Age

>> ------- ------------- -----------

Ostrya 100 20

Fagus 100 20

SiteSelection Complete

CohortsRemoved SpeciesList

>> Species Cohorts removed

>> ------- ---------

Ostrya 20-40

Fagus 1-3 20-40

Castanea 1-3

Quercus_cer 1-6

Quercus_pub 1-6

Abies 1-6

Acer 1-6

Fraxinus 1-6

Prescription ConifersReduction

StandRanking Economic

>> Species Economic Rank Minimum Age

>> ------- ------------- -----------

Pinus_nig 80 20

Pseudotsuga 100 20

Abies 100 1

SiteSelection Complete

CohortsRemoved SpeciesList

>> Species Cohorts removed

>> ------- ---------

Pinus_nig All

Pseudotsuga All

Abies All

Castanea 1-3

Fagus 1-3

Quercus_cer 1-6

Quercus_pub 1-6

Acer 1-6

Fraxinus 1-6

>>-----------------------------------------------------------------

>> PRESCRIPTIONS FOR "SELECTION" MANAGEMENT AREA

Prescription ConifersEradication

StandRanking Economic

>> Species Economic Rank Minimum Age

>> ------- ------------- -----------

Abies 100 20

Pinus_nig 80 20

Pseudotsuga 100 20

SiteSelection Complete

CohortsRemoved SpeciesList

>> Species Cohorts removed

>> ------- ---------

Abies All

Pinus_nig All

Pseudotsuga All

Castanea 1-3

Fagus 1-3

Quercus_cer 1-6

Quercus_pub 1-6

Acer 1-6

Fraxinus 1-6

Prescription ThinnCut

StandRanking Economic

>> Species Economic Rank Minimum Age

>> ------- ------------- -----------

Castanea 100 40

Fagus 100 40

Ostrya 60 40

Quercus_cer 100 40

Quercus_pub 100 40

Pseudotsuga 60 1

SiteSelection Complete

CohortsRemoved SpeciesList

>> Species Cohorts removed

>> ------- ---------

Castanea 1-3 40-50

Fagus 1-3 40-90

Ostrya 40-60

Quercus_cer 1-6 40-80

Quercus_pub 1-6 40-90

Pseudotsuga All

Abies 1-6

Acer 1-6

Fraxinus 1-6

Prescription SelectCut

StandRanking Economic

>> Species Economic Rank Minimum Age

>> ------- ------------- -----------

Castanea 100 50

Fagus 100 90

Ostrya 60 60

Quercus_cer 100 80

Quercus_pub 100 90

Pseudotsuga 60 1

SiteSelection Complete

CohortsRemoved SpeciesList

>> Species Cohorts removed

>> ------- ---------

Castanea 1-3 50-100

Fagus 1-3 90-140

Ostrya 60-150

Quercus_cer 1-6 80-200

Quercus_pub 1-6 90-200

Pseudotsuga All

Abies 1-6

Acer 1-6

Fraxinus 1-6

>>-----------------------------------------------------------------

>> PRESCRIPTIONS FOR "NO PRESCRIPTION" MANAGEMENT AREA

Prescription AbiesEradication

StandRanking Economic

>> Species Economic Rank Minimum Age

>> ------- ------------- -----------

Abies 100 1

SiteSelection Complete

CohortsRemoved SpeciesList

>> Species Cohorts removed

>> ------- ---------

Abies All

Castanea 1-3

Fagus 1-3

Quercus_cer 1-6

Quercus_pub 1-6

Acer 1-6

Fraxinus 1-6

>>-----------------------------------------------------------------

>> PRESCRIPTIONS FOR UNGULATE IMPACT

Prescription Ungulate

StandRanking Economic

>> Species Economic Rank Minimum Age

>> ------- ------------- -----------

Castanea 80 1

Fagus 60 1

Quercus_cer 100 1

Quercus_pub 100 1

Abies 80 1

Acer 100 1

Fraxinus 100 1

SiteSelection Complete

CohortsRemoved SpeciesList

>> Species Cohorts removed

>> ------- ---------

Castanea 1-3

Fagus 1-3

Quercus_cer 1-6

Quercus_pub 1-6

Abies 1-6

Acer 1-6

Fraxinus 1-6

>>-----------------------------------------------------------------

>> PRESCRIPTION IMPLEMENTATION

HarvestImplementations

>> Mgmt Area Prescription Harvest Area Begin Time End Time

>> --------- ------------ ------------ ---------- --------

1 QuercusCoppice 4.3%

1 CastaneaCoppice 1.7%

1 BroadleavesCoppice 1.3%

1 ConifersReduction 5%

2 ConifersEradication 0.9%

2 ThinnCut 1%

2 SelectCut 1%

4 AbiesEradication 100%

1 Ungulate 100%

2 Ungulate 100%

4 Ungulate 100%

>>-----------------------------------------------------------------

>> OUTPUTS

PrescriptionMaps harvest/prescripts-{timestep}.gis

EventLog harvest-event-test-log.csv

SummaryLog harvest/summary-log.csv

# Species

LandisData Species

>> Sexual Shade Fire Seed Disperal Dist Vegetative Sprout Age Post-Fire

>> Name Longevity Maturity Tol. Tol. Effective Maximum Reprod Prob Min Max Regen

>> -------------------- --------- -------- ----- ---- --------- ------- ----------- ---- ---- ----------

Quercus_cer 350 40 2 3 20 40 0.9 1 80 resprout

Fagus 300 60 5 2 25 50 0.75 1 100 resprout

Castanea 600 15 3 2 20 30 0.9 1 600 resprout

Quercus_pub 350 50 2 3 20 40 0.75 1 80 resprout

Pinus_nig 300 30 2 3 40 100 0 0 0 none

Abies 300 70 4 3 50 160 0 0 0 none

Ostrya 150 30 4 2 80 180 0.8 1 100 resprout

Pseudotsuga 700 20 3 5 100 250 0 0 0 none

Pinus_pin 200 15 1 4 40 300 0 0 0 none

Fraxinus 200 20 3 2 50 140 0.8 1 80 resprout

Robinia 100 15 2 1 30 120 0.9 1 100 resprout

Acer 300 15 4 2 100 300 0.8 1 100 resprout
