# Supplementary figures and images for: Effects of forest management and roe deer impact on a mountain forest development in the Italian Apennines: A modelling approach using LANDIS-II
Source: PLoS One. 2019 Nov 6;14(11):e0224788. doi: 10.1371/journal.pone.0224788 (PMC6834274; doi:10.1371/journal.pone.0224788)

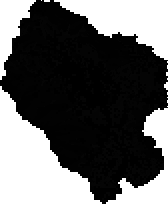

Supplement: S5 File — (TIF) [file pone.0224788.s005.tif]
